# Robust Stick‐and‐Play Photothermal Icephobic Film with Bioinspired Insulation Cells

**DOI:** 10.1002/advs.202502687

**Published:** 2025-03-16

**Authors:** Xiaohu Xia, Haotian Chen, Yiming Wang, Haidong Yu, Bingsuo Zou, Yabin Zhang

**Affiliations:** ^1^ State Key Laboratory of Featured Metal Materials and Life‐cycle Safety for Composite Structures Guangxi Key Laboratory of Processing for Non‐ferrous Metals and Featured Materials, MOE Key Laboratory of New Processing Technology for Nonferrous Metals and Materials Guangxi University Nanning 530004 P. R. China

**Keywords:** anti‐/de‐icing, bioinspired insulation cells, durability, stick‐and‐play, thermal management film

## Abstract

The integration of photothermal de‐icing and micro/nanostructured anti‐icing technologies into a surface is regarded as a promising solution to solve ice accretion aggravated. Unfortunately, light‐dependent heat effect and large‐scale production of micro/nano building still challenge the anti‐icing ability and applications for real‐world. Herein, a stick‐and‐play film embedded with bioinspired thermal‐management cells is developed. Inspired by the hollow framework of lotus seedpods, thermal‐management hydrophobic microcells (THMC) are designed by incorporating candle soot into insulating porous diatomite. Once embedding such microcells into PDMS substrate, the resulting THMC film delivers effective photothermal effect since the synergy of photothermal and insulation design. COMSOL simulations demonstrate thermal management effect of the “framework” and “photothermal seeds,” which causes increases in the heating rate by 20% and equilibrium temperature by 10%. Moreover, the self‐similarity structure of THMCs enable them to have durable hydrophobicity (148.7°) and photothermal effect (79.9 °C) even after repeated abrasions. When a stick‐and‐play functionality is imparted by designing adjustable milli‐suction cups, this THMC film could adhere effectively to various surfaces despite dry and humid conditions while maintaining efficient anti‐/de‐icing capabilities. This study provides a designing strategy of robust and efficient photothermal films constructed from THMC and finds flexible use for diverse surfaces.

## Graphical abstract

Inspired by the seed‐and‐framework structure of lotus seedpods, a photothermal anti‐icing film is developed by embedding thermal‐management hydrophobic microcells (THMC) into PDMS. Combining photothermal/insulating synergy and hydrophobic microcells, it achieves heat accumulation, surface adhesion, and robust anti‐icing under weak light, enabling scalable fabrication for industrial and daily icing‐resistant applications.

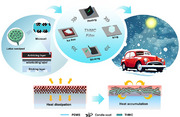

## 1 Introduction

The phenomenon of surface icing accretion has brought about a range of inconveniences in diverse scenes of human production and activities, even causing enormous economic losses and provoking catastrophic events in aviation, shipping, transportation, industrial, and energy production.^[^ ^1^, ^2^ ^]^ With frequent occurrence of extreme weather like freezing rain and snowstorms in cold climates, exemplified by a large‐scale low temperature freezing disaster occurred in southern China in 2022, dealing with the exacerbated icing accretion has become imperative. Accordingly, a large variety of measures have been developed and employed to try to mitigate the impact of undesired ice accretion on infrastructures and transportations in a large variety of scenarios.^[^ ^3^ ^]^ These anti‐/de‐icing methods mainly fall into active and passive strategies. Active strategy refers to the effective removal of ice layers accumulated through chemical treatment, mechanical vibration, electric heating, wind heating, and so on. However, they overly depend on energy input from external systems and have drawbacks of environmental pollution, complex operations, large energy consumption, and low efficiency.^[^ ^4^, ^5^ ^]^ Passive method utilizes icephobic surfaces with hydrophobicity and low adhesion to repel droplets before freezing, delay freezing of water droplets, and make the shedding of the ice layer spontaneously after freezing, among which superhydrophobic surfaces have gained great attention for anti‐icing.^[^ ^6^, ^7^, ^8^ ^]^ Nevertheless, such a passive anti‐icing method poses high requirements for the durability and large‐scale fabrication of surface structures, especially with micro/nano scales. Their prolonged exposure to low‐temperature environments often leads to inevitable frost formation.^[^ ^9^ ^]^ Therefore, the combination of active and passive strategies, which complements their respective deficiencies, is deemed a more promising avenue.

The integration of various photothermal or electrothermal materials into superhydrophobic surfaces has proven to be effective for synergistically enhancing anti‐/de‐icing performance.^[^ ^10^ ^]^ Recent studies have demonstrated that photothermal anti‐icing technology can be effectively implemented when utilizing solar energy. Various photothermal materials have been introduced into a wide range of anti‐/de‐icing applications. Given high photothermal conversion efficiency, inexpensiveness, and availability of candle soot (CS), an efficient superhydrophobic photothermal surface has been achieved based on it.^[^ ^11^ ^]^ Such a surface not only disables the formation of ice even at the environmental temperature of as low as −50 °C but also melts the accumulated ice rapidly. In view of self‐cleaning ability of superhydrophobic surfaces for the self‐removal of the melted water and contaminants, a clean and dry surface is anticipated during the photothermal process, which conduces to efficient light absorption for high photothermal conversion. This feature has been employed to construct an effective solar anti‐icing/frosting surface with condensate self‐removing ability.^[^ ^12^ ^]^ The synergistic effect of enhanced condensate self‐removal and efficient solar anti‐icing prevents the ice accretion even at an ambient environment of −50 °C and extremely high humidity. Despite numerous advantages demonstrated by the integration of photothermal capabilities with superhydrophobicity constructed from micro/nanostructures, the enhanced performance is still needed in different practical scenarios, especially in weak‐light and no‐light settings. On the basis of the photothermal mechanism, photothermal icephobic ability is significantly determined by the interaction of light with engineering surface structures and the full utilization of the generated heat.^[^ ^13^ ^]^ Accordingly, various approaches have been developed, including the utilization of low‐emissivity materials to enhance thermal conversion efficiency under reduced illumination,^[^ ^14^ ^]^ the introduction of phase change materials for effective heat management,^[^ ^15^ ^]^ and the combination of supplementary energy like electricity to facilitate multi‐modal coordinated operation.^[^ ^16^ ^]^ Unfortunately, to achieve satisfied photothermal anti‐icing efficiency is limited through the combination of these technologies without the exhaustive consideration of light absorption and thermal management for high‐efficient photothermal anti‐/de‐icing ability.

Surface engineering is pivotal for the realization of light absorption, thermal management, and superhydrophobicity, especially micro/nanoscale building blocks. On one hand, surface micro/nanostructures and their trade‐off for light absorption and superhydrophobicity are critical between for the performance improvement. On the other hand, these structures are fragile and susceptible to collapse or damage under external stresses, whose poor mechanical durability is detrimental to anti‐icing capabilities and long‐term service, thereby severely impeding further real‐world applications.^[^ ^17^ ^]^ To improve the anti‐/de‐icing ability and robustness simultaneously, various strategies should be considered and leveraged in the process of surface structure design, including the self‐similar strategy that ensures damaged structural blocks retain similarity or sufficient functionality with undamaged counterparts, and the protective strategy that entails constructing multi‐layered architectures utilizing larger and more robust structures to safeguard smaller and more delicate components. Nevertheless, self‐similar strategy merely evades issues related to structural wear and its efficacy is contingent upon structural height.^[^ ^18^, ^19^ ^]^ The protective strategy requires quite strict requirements in the overall structural design, facing challenges associated with complex processes and elevated costs.^[^ ^20^, ^21^ ^]^ Consequently, a novel cellular design concept was proposed that harmoniously integrates structural integrity and functional resilience within a single coating layer.^[^ ^22^ ^]^ Such a structure engineering strategy involves the meticulous design of fundamental cells, consisting of rigid microshells and detachable nanoseeds. The introduction of a unique framework and self‐similar hierarchical micro/nanostructures enables the surface to exhibit exceptional anti‐abrasion properties. With the complexation and diversity of application scenarios, current technologies that mainly concentrate on individual application scenarios are unsatisfactory, which neglect universal adaptability across diverse surfaces in a wide range of scenarios. Therefore, the realization of micro/nanoscale building blocks over large area and different materials is critical and poses challenges for the large‐scale applicability of photothermal anti‐icing surfaces with both robustness and high photothermal ability.^[^ ^23^ ^]^

**Scheme 1:**
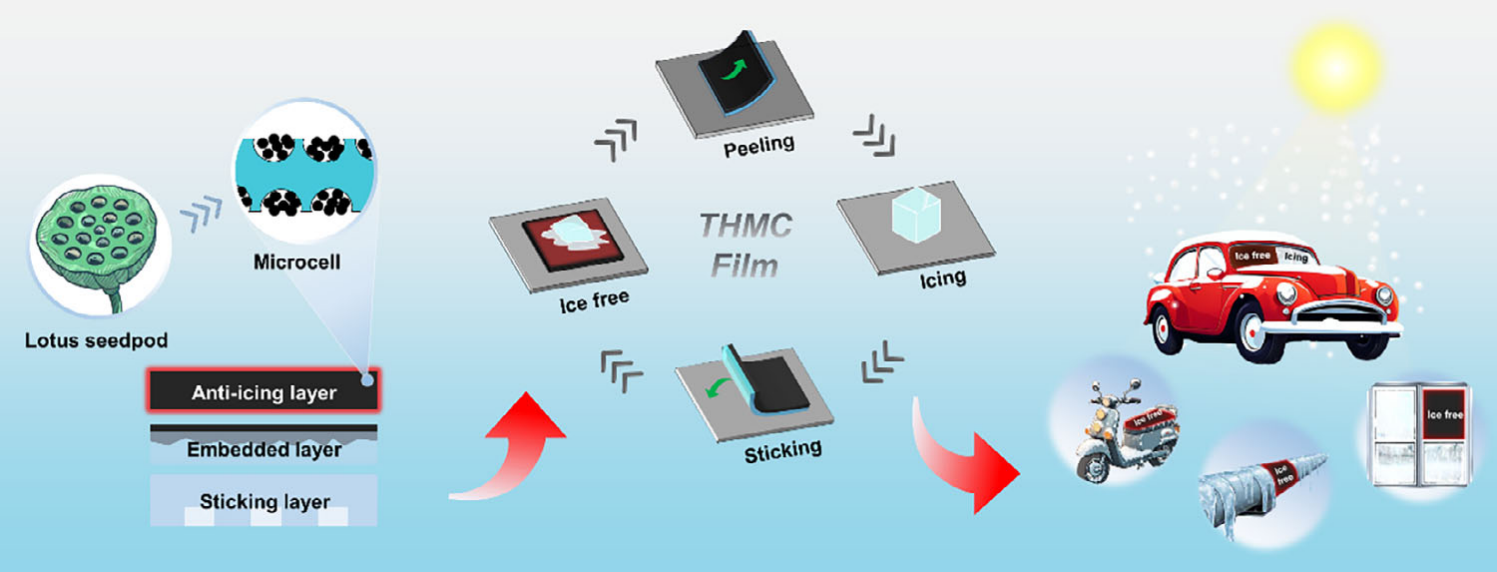
An innovative photothermal anti‐/de‐icing film embedded with lotus‐seedpod‐inspired thermal‐management units featuring a stick‐and‐play capability that facilitates the efficient and rapid removal of ice from diverse surfaces in various icing scenarios of industrial and human activities.

Given the photothermal improvement limitation from the material perspective and various shape surfaces in real settings, an innovative photothermal anti‐/de‐icing film featuring a stick‐and‐play capability is developed based on the surface engineering of thermal management to achieve anti‐/de‐icing on diverse surfaces (**Scheme** **1**). To improve the durability of building blocks, a THMC with armor architecture and a hierarchical self‐similarity structure is designed by incorporating nanoscale candle soot (CS) possessing high photothermal conversion efficiency into porous insulating microscale diatomite, drawing inspiration from the seed‐and‐framework structure of lotus seedpods. When incorporating THMCs onto a PDMS substrate, the THMC films are produced demonstrating outstanding hydrophobic characteristics and high photothermal conversion efficiency. Analogous to the hollow framework found in lotus seedpods, the use of diatomite modifies the thermal conduction process on the THMC film. It enables the surface temperature of the THMC film to reach 84.2 °C at room temperature. As demonstrated by COMSOL numerical simulation, a pronounced heat accumulation effect is observed, which significantly enhances the photothermal conversion efficiency of THMCs, thereby enabling effective photothermal de‐icing ability within 377 s. Due to the presence of armor architecture and hierarchical self‐similar structure, the THMC film is capable of effectively preserving its surface hydrophobicity and photothermal conversion efficiency even after abrasion, thereby augmenting its practical applicability. When adjustable milli suction cups are incorporated onto the reverse side of the THMC film, a stick‐and‐play functionality is endowed with the film. This feature allows the film to effectively adhere to various surfaces despite dry and humid conditions while also imparting anti‐icing and de‐icing capabilities. Such stick‐and‐play THMC films can deliver efficient anti‐icing and defrosting capabilities across diverse surfaces through a streamlined standardized preparation process, thereby demonstrating significant potential in various icing scenarios within both industrial and human activities.

## 2 Results and Discussion

### 2.1 Design and Fabrication of the THMC Film

The lotus seedpod is composed of lotus seeds embedded within a pod structure. In nature, the framework of the lotus pod functions to protect and nourish the seeds. Further, the pod components that constitute the lotus seedpod are permeated with abundant hollow and porous structures, as illustrated in Figure S1 (Supporting Information). It has been demonstrated that such structures possess potential thermal management capabilities.^[^ ^24^, ^25^ ^]^ In this regard, inspired by the hollow framework structure of lotus pods for breeding seeds, the utilization of porous microscale structure as the framework to load nanoparticles as seeds is anticipated to construct pod‐like microcells with armor architecture and hierarchical structures.

**Figure 1:**
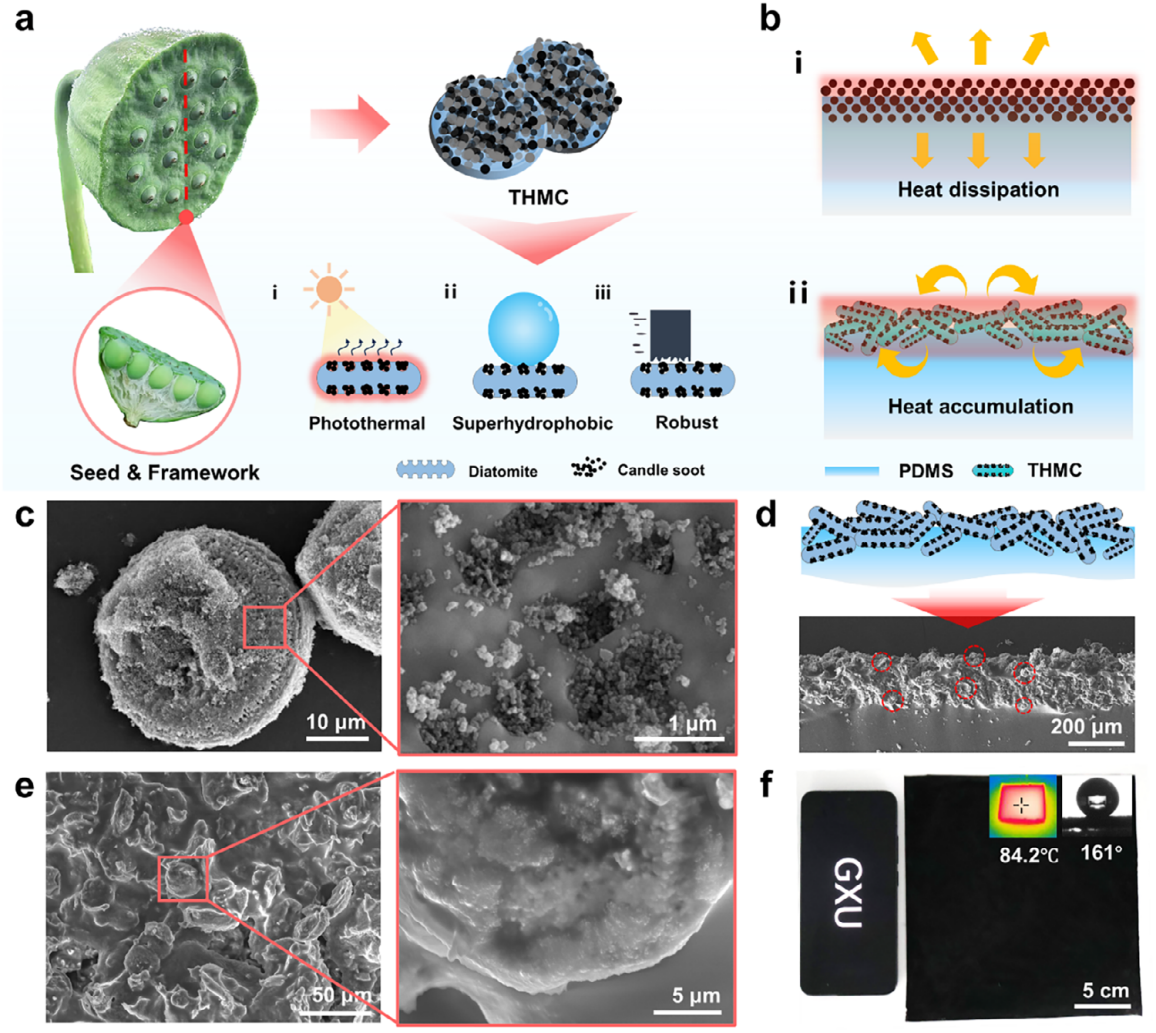
Structure and performance characterization of the THMC film. a) Schematic depicting the integration of a microscale diatomite framework with CS nanoparticles to achieve enhanced photothermal properties, hydrophobicity, and durability. b) Schematic diagram of thermal management effect of a THMC. c) SEM images illustrating the distribution of CS within the THMC with the CS to diatomite ratio of 1:3. d) Schematic diagram representing the embedding structure of the THMCs onto the film. e) SEM images of the THMC film surface featuring an embedded architecture. f) Optical images of the THMC film with optimal spraying thickness, showing a contact angle of 161° and surface temperature of 84.2 °C when exposed to one‐sun irradiation at room temperature.

After billions of years of evolution, nature provides a wealth of microscale templates. As a well‐known microorganism, the diatomite composed of silica shows diverse morphology and porous structures with a few hundred nanometers in diameter (Figure S2a, Supporting Information), which enable it to have the ability of adsorption, non‐flammability, permeability, sound‐proof, water‐proof and heat‐insulation.^[^ ^26^, ^27^, ^28^ ^]^ These features, together with micron size and easy accessibility, make the diatomite become an ideal candidate support for carrying various types of nanomaterials to achieve different applications. It has been reported that CS exhibits excellent photothermal conversion efficiency and strong hydrophobicity.^[^ ^29^ ^]^ This low‐cost carbon nanomaterial possesses a chain‐like nanostructure (Figure S2b, Supporting Information) with an average diameter of 30–50 nm and abundant hydrophobic alkane groups from paraffin burning, accompanied by poor durability in extreme settings.^[^ ^30^ ^]^ When the silica diatomite was leveraged to load CS nanoparticles, the synergy of micro/nanostructure and multiple components enables the formed CS@diatomite microcell to exhibit photothermal, hydrophobic, and robust properties, as illustrated in **Figure** **1**a. Eventually, it is anticipated that the introduction of THMCs into the film would modify the heat transfer efficiency, resulting in a notable heat accumulation on the film surface when compared to films composed solely of CS, as illustrated in Figure 1b.

**Figure 2:**
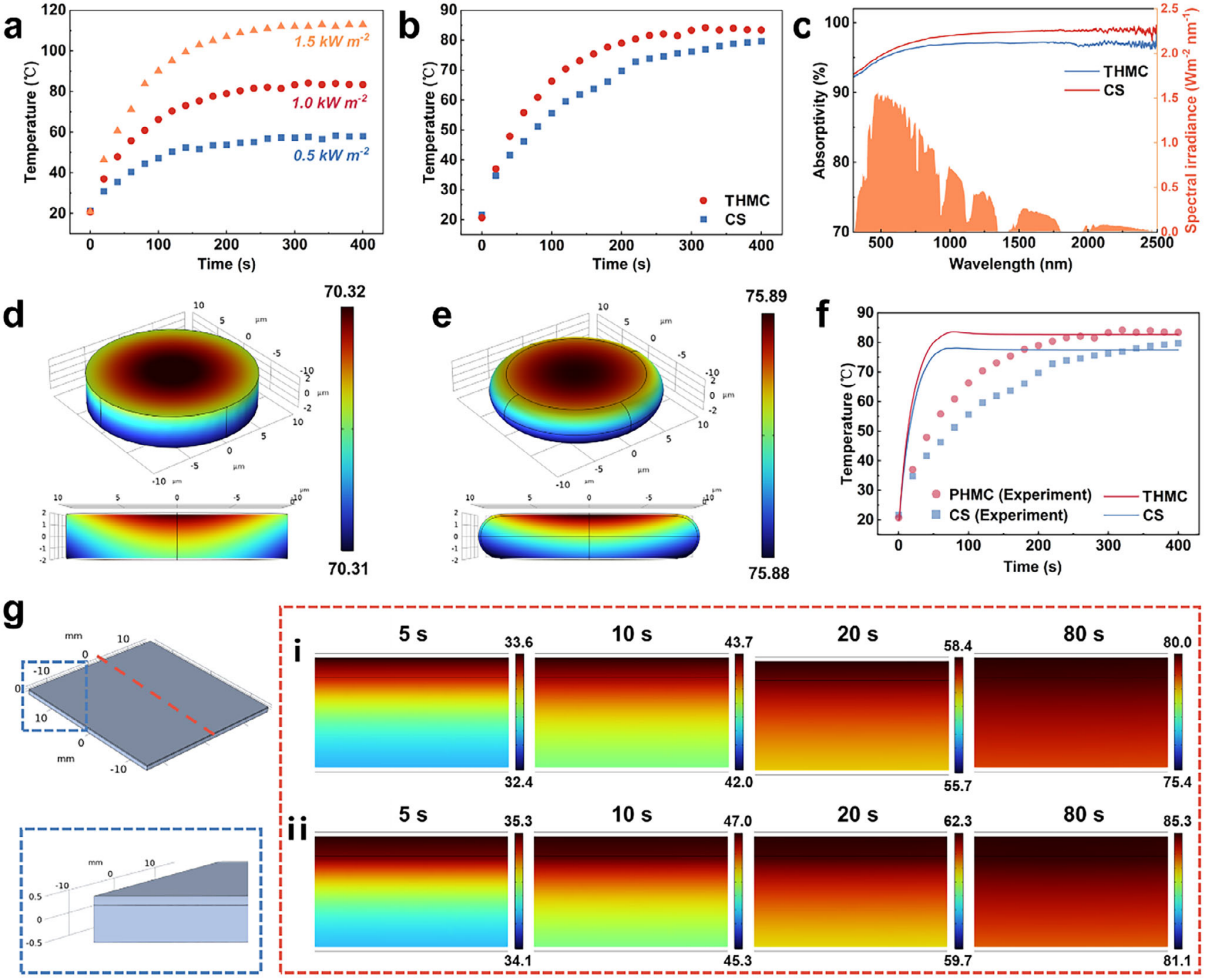
Photothermal enhancement mechanism of the THMC film. a) Surface temperature changing curves of the THMC film over time under different light intensities. b) Surface temperature variation of the THMC and CS films over time under light of 1 kW m^−2^ at room temperature. c) Solar absorptivity curves of THMC and CS films. COMSOL simulation of temperature distribution of the d) CS cell and e) THMC under one sun irradiation. f) Temperature–time curves of CS and THMC films based on COMSOL model and real tests. g) COMSOL simulation of the temperature temporal variation of the cross‐section of a CS film under sunlight exposure, where high‐temperature regions in the THMC film model are obvious.

To load CS nanoparticles onto the diatomite efficiently, the dispersed solution of CS and diatomite is vigorously stirred to ensure complete impregnation of soot into diatomite, followed by vacuum drying to yield the THMCs (**Figure** **2**c). With the THMCs sprayed onto a semi‐polymerized PDMS substrate, a composite THMC film is formed, featuring an embedded structure that significantly enhances the robustness of THMC inside the PDMS substrate. As the ratio of CS to diatomite varies from 1:5 to 1:1, THMCs with various morphologies show different embedding forms onto the film (Figure 1c and Figure S3, Supporting Information). Moreover, THMC films fabricated at different ratios present relatively consistent photothermal performance (Figure S4a, Supporting Information). This is because the content of CS in these films is sufficient, and its slight variations do not result in significant temperature differences within the films. With the CS reducing, the THMC film exhibits a considerable decrease in hydrophobicity compared with the one with more CS content (Figure S4b, Supporting Information). This might arise from the insufficient coverage of CS at lower ratios on the surface THMCs, resulting in an uneven distribution of nanostructure on the film surface. Accordingly, the THMC film with the CS to diatomite ratio of 1:3 is fixed for the following characterization and discussion.

Considering the polymerization state of the PDMS film in determining the ultimate surface morphology of the film,^[^ ^31^ ^]^ the influence of the PDMS polymerization state on film performance was examined. THMC films synthesized in the semi‐polymerized state demonstrate embedding architectures (Figure 1d), characterized by a diverse array of micro/nanostructures on their surface (Figure 1e). Under low polymerization conditions, THMCs are driven by gravity to gradually submerse into the PDMS substrate, thereby impeding the formation of an effective microstructured surface (Figure S5a,b, Supporting Information). At the fully polymerized state, the THMC is entirely exposed on the surface of the PDMS substrate, thus making the film exhibit a complex structure (Figure S5c,d, Supporting Information). Such a THMC film features an embedded structure and demonstrates markedly improved photothermal performance and hydrophobicity compared to other configurations (Figure S6, Supporting Information). Subsequently, THMC films were prepared with of different thicknesses displaying hydrophobicity and photothermal performances. With the thickness rising, the photothermal effect of the THMC film surface progressively enhances (Figure S7a, Supporting Information). However, when the thickness of the THMC layer exceeds a threshold of 150 µm, the temperature remains constant at about 84 °C, without significant improvement in the photothermal effect. Concurrently, an increase in THMC layer thickness enhances hydrophobicity, which achieves a maximum hydrophobicity of 161.3° at 200 µm (Figure S7b, Supporting Information). This is primarily attributed to the uneven distribution of THMC in a short coating time. So, the optimal thickness of the THMC layer is fixed to 200 µm, enabling the film to present a high photothermal temperature of 84.2 °C and good superhydrophobicity with a contact angle (CA) of 160° and a sliding angle (SA) of 5°, as given in Figure 1f. Furthermore, the high photothermal temperature is reached from room temperature to 84.2 °C within 360 s under one‐sun irradiation, suggesting a good photothermal heating rate superior to ones reported previously.^[^ ^32^, ^33^, ^34^, ^35^, ^36^ ^]^ Besides, this high hydrophobicity of the THMC film could be extended to a range of liquids with different surface tensions, demonstrating high lyophobic behavior towards these liquids (Figure S8, Supporting Information). Despite CAs with less than 150° observed on the film surface, their SAs remain below 10°, indicating low adhesion of the THMC film for the rapid detachment of drops. This is further supported by the fact that liquid droplets can effortlessly roll off a cover slip inclined at 45° (Figure S9, Supporting Information), thereby efficiently removing dust particles. In contrast, residual water and dust particles are observed on the surface of pure PDMS films. These results all verify the success in the design and preparation of the THMC film with the exceptional superhydrophobicity and remarkable photothermal performance.

### 2.2 Photothermal Enhancement Ability from Bioinspired Insulating Cells

As expected, the incorporation of THMCs with intricate structure design and good thermal‐management ability would promote a high surface temperature of the film under light irradiation. Indeed, the THMC film exhibits exceptional photothermal temperature under different light intensities over time, as illustrated in Figure 2a. At a low illumination intensity of 0.5 sun, the film achieves a surface temperature of 58.3 °C within a short period (200 s), which is beneficial for anti‐/de‐icing. With the solar irradiation intensity equivalent to one‐sun at room temperature, the film surface temperature rises to 84.2 °C (Figure 2b). Compared with that of the pure CS film, the photothermal conversion efficiency has increased by 6%. Meanwhile, THMC films demonstrate a faster heating rate, exhibiting an increase of approximately 20% compared to that of CS films, suggesting the synergistic thermal management of the insulating diatomite and photothermal CS within the hierarchical structure. To figure out the potential thermal‐management mechanism of their cooperation, detailed exploration and analysis were carried out. It has been found that the fundamental theoretical descriptions for photothermal conversion involve light harvesting, light‐to‐heat conversion, and heat transfer. Considering the importance of light capturing, which indicates how photothermal materials absorb incident photon energy effectively,^[^ ^37^ ^]^ light absorption rates of the as‐obtained films were evaluated. As shown in Figure 2c, the THMC film with a high photothermal temperature counterintuitively exhibits a lower light absorption rate of 96.5% than the CS film (97.9%) with a lower temperature. This discrepancy in light absorption might arise from the jagged structure of aggregated CS nanoparticles, thereby promoting sunlight capturing through multiple internal reflections and enhancing the efficiency of solar energy absorption.^[^ ^11^ ^]^ When CS nanoparticles are loaded onto microscale diatomite despite of successful construction of hierarchical structure, the lack of light absorption capability in diatomite ultimately results in a reduction in the light harvesting capacity of THMC. To elucidate why the THMC film with low light absorption exhibits a superior photothermal effect, light‐to‐heat conversion and heat transfer involved in the photothermal conversion process are taken into account. Because of the same CS nanoparticles used as photothermal materials, it is essential to elucidate the reason for the THMC film showcasing superior photothermal effect from a heat transfer perspective.

In general, heat transfer occurs through three primary mechanisms: conduction, convection, and radiation, driven by thermal gradients. Theoretically, the total energy absorbed by the film is balanced by the total energy dissipated,^[^ ^38^ ^]^ as expressed in the following equation.

(1) $$Q_{\mathrm{sun}}=Q_{\mathrm{cond}}+Q_{\mathrm{conv}}+Q_{\mathrm{rad}}$$

where *Q*_rad_ *, Q*_conv,_ and *Q*_cond_ are the heat loss due to thermal radiation, convection, and conduction, respectively. Under the condition of ensuring identical external environments, differences in thermal convection can be neglected. Furthermore, due to the low power of thermal radiation and the small area of the thin film used for testing, the minor variations in thermal radiation play a negligible role in the overall heat transfer process. Therefore, without the consideration of convection and radiation, the above formula is derived:

(2) $$Q_{\mathrm{sun}}=Q_{\mathrm{cond}}=kA\frac{\left(T_{1}-T_{2}\right)}{L}$$

where *k* is the thermal conductivity of the material, *A* is the surface area of heat transfer, *T*_1_ and *T*_2_ are the steady state temperatures of two different films, and *L* is the thickness of the medium. According to Equation 2, the variation in thermal conductivity is the primary factor contributing to the significant change in *ΔT* observed. To further clarify the underlying thermal‐management mechanism, a transient heat transfer model was implemented in COMSOL software for the films (detailed explanations in Supporting Information). Two microscale unit models of comparable dimensions (20 µm × 20 µm) are established initially in light of the uniformity of films and the simplification of simulation. One micromodel composed entirely of CS nanoparticles is established to manifest pure film (Figure 2d), and another consists of CS‐loaded diatomite to represent the THMC film (Figure 2e). When exposed to solar radiation, surface temperatures of both microcells are recorded at 70.3 °C for the CS microunit and 75.9 °C for the THMC one, which is consistent well with the trend of experimental results. Notably, the temperature exhibits an inconsistent distribution near their upper surface, whose distribution within the THMC microcell is more concentrated in the area exposed to sunlight. To verify the effectiveness of microunit simulations, a model consisting of a microcell structure is taken from the part of the film (20 mm × 10 mm) (Figure S10a,b, Supporting Information). After the thermal field simulation, it is observed that the high‐temperature region of the THMC film becomes increasingly concentrated near its surface embedded by THMCs, whereas the temperature distribution on the CS film appears more dispersed (Figure S10c,d, Supporting Information). This centralized surface thermal effect results in an accelerated heating rate and a higher temperature for the THMC film compared with those of the CS film (Figure S10e, Supporting Information), further demonstrating the improved photothermal effect of the THMC film.

Given the complicacy in simulation calculation of the heat transfer process of the films with complex surface structures, a simplified macro‐model of the CS film and THMC film was established based on the above simulation results of the microunits, where both simulation models comprise a photothermal layer and a transparent PDMS layer. The model surface is heated under constant solar irradiation. The maximum temperatures recorded for the pure CS and THMC film models are 78.1 °C and 83.6 °C, respectively, which agrees with experimental data well (Figure 2f). Furthermore, during the heating and stabilization phase of the film model, it was distinctly observed that high‐temperature regions in the THMC film model are concentrated on its surface embedded with photothermal materials, indicating significant heat accumulation on the surface (Figure 2g). This phenomenon could be attributed to the incorporation of diatomite, which further reduces the thermal conductivity of the photothermal conversion layer on the film surface. Consequently, the heat exchange efficiency between the film surface and the air decreases, leading to a further increase in the equilibrium temperature of the film surface. Based on these analyses, the concept of combining photothermal CS with insulating porous diatomite significantly promotes heat accumulation, thereby enhancing the photothermal effect of the films. By making full use of synergistic effect of photothermal and insulating materials based on thermal management, the surface temperature of photothermal films can be effectively elevated under low‐temperature and low‐illumination conditions, which holds great promise in anti‐/de‐icing applications.

### 2.3 Stick‐and‐Play Functionality from Structural Bioinspiration

**Figure 3:**
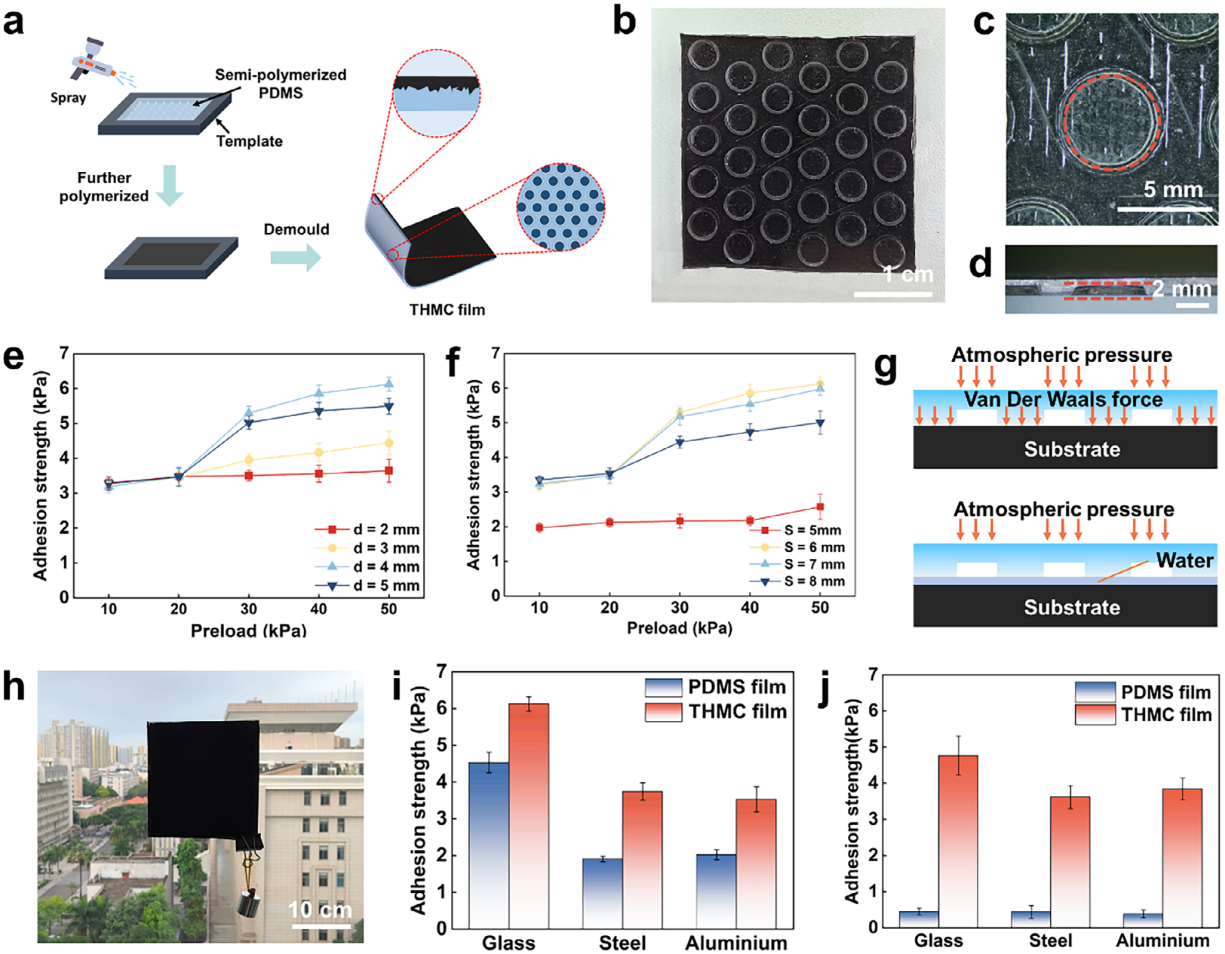
Stick‐and‐play property of the THMC film. a) Schematic representation of the preparation for suction cups on the THMC film. b) Optical image of suction cup structures on the THMC film, along with an enlarged view (c). d) Sectional image of the THMC film, showing the suction cup structure of 0.6 mm in depth. Changing curves of adhesion force versus preload of the THMC film with e) different diameters and f) spacings of suction cups. g) Schematic diagram illustrating the adhesion mechanism of THMC film. h) Digital photos of the film adhering to the building glass, showing strong adhesion even if being stretched by 200 g weight. Adhesion of the film to surfaces of commonly engineering materials under i) dry and j) wet conditions (load of 50 kPa).

Given the convenience of instant‐use application onto curved or flat surfaces as well as the scalability of fabrication, a stick‐and‐play functionality is imparted into the THMC film for repeated adhesion and detachment on a wide range of surfaces by fabricating adjustable milli‐scale suction cups on the reverse side of the THMC film using a templating method (**Figure** **3**a). As shown in Figure 3b–d, the resultant suction cup on the THMC film, inspired by octopus suckers, is a hexagonal arrangement of cylindrical pits that has a diameter of 4 mm, a depth of 0.6 mm, and a center‐to‐center spacing of 6 mm. Such a structural design is favorable for the optimization of surface contact area while minimizing air trapping during application, which could enhance adhesion performance. To achieve desired stick‐and‐play adhesion, the effects of structural parameters of the suction cup array on the THMC film were further explored using a home‐made device designed for precise measurement under controlled conditions (Figure S11, Supporting Information). As given in the Figure 3e,f, under dry conditions, different pre‐stresses applied to the film result in different normal adhesion forces for the film adhering to a smooth glass plate. Normal adhesion forces increase with the increase of the preload for all samples. The increase of force with the increases in preload should be attributed to the effective contact area of the interacting surface, which also increases to a stable value with the increase in preload.^[^ ^39^ ^]^ It is reported that adhesion forces are usually originated from two aspects.^[^ ^39^ ^]^ One is the van der Waals forces generated by molecular interfacial interactions between PDMS and substrate, whereas the other is atmospheric pressure exerted by the geometric structure and configuration of the suction cup. Due to the synergy of two forces, effective adhesion of the THMC film to glass surfaces is achieved without detachment under lower loads (Figure 3g). Meanwhile, proper adhesion strength permits easy removal of THMC films under minimal external forces, which is an essential characteristic for their large‐area and instant application scenarios like vehicle windshield. As demonstrated by the adhesion of the THMC film to a glass surface (Figure 3h), the THMC film keeps secure attachment onto the glass surface even under a load of 200 g, further supported by the similar adhesion to other surfaces like copper, steel, aluminum, PMMA, and ceramics in Figure S12a–f (Supporting Information). These results verify the effective adhesion of THMC film to a myriad of surfaces. Due to the intrinsic high tangential friction of PDMS materials on these surfaces, the films, with an effective adhesion area of approximately 20 mm × 20 mm, still sustain a tangential load of 400 g (Figure S11g,h, Supporting Information). Furthermore, its lower adhesion strength, below 10 kPa, facilitates easy instant removal from the surface.

**Figure 4:**
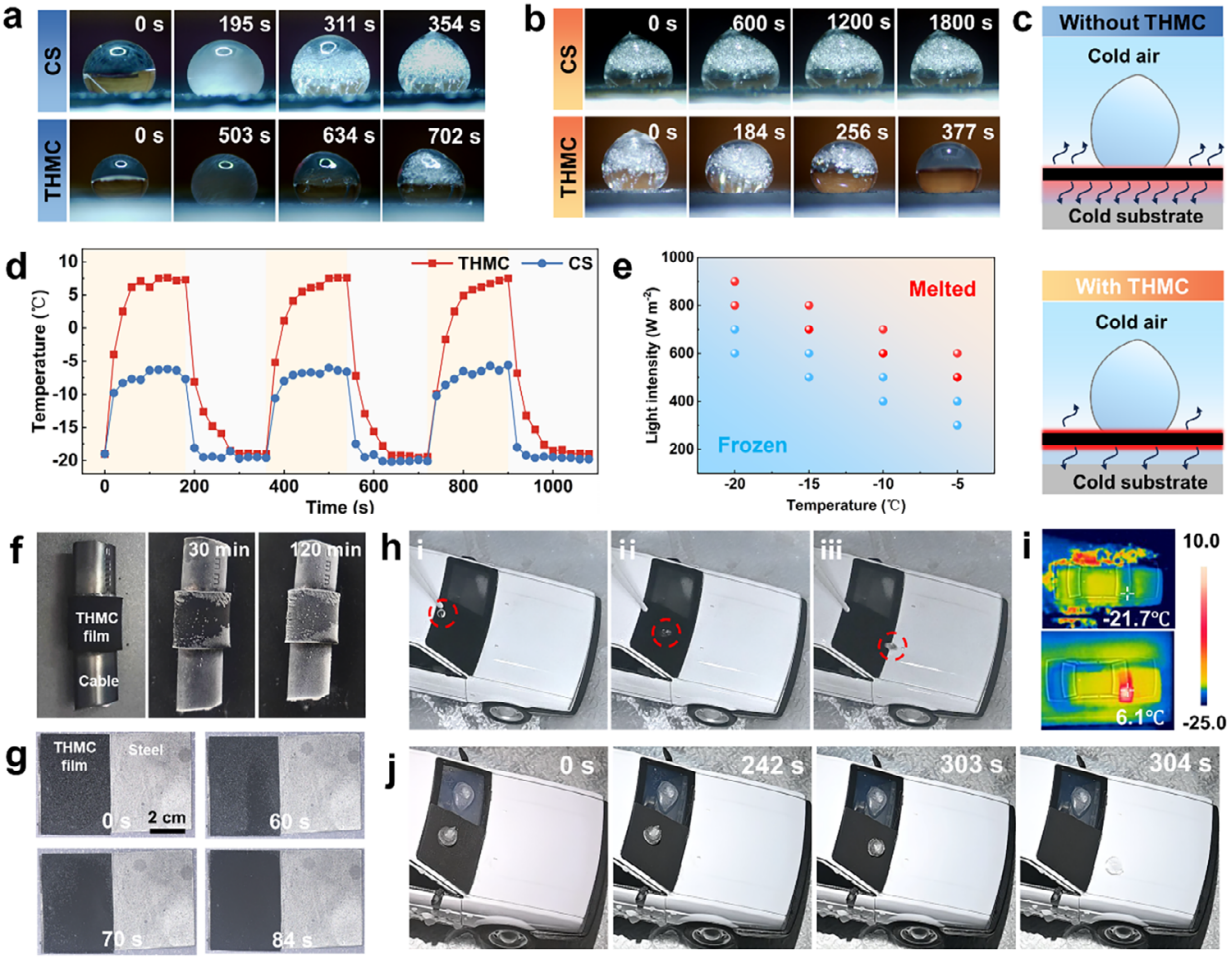
Efficient anti‐/de‐icing of the THMC film. a) Icing delay process of droplets on the surfaces of CS and THMC films. b) Melting process of a frozen droplet on the surfaces of CS and THMC films under one‐sun irradiation. c) Schematic diagram illustrating the process of removing liquid droplets via photothermal effect on diverse surfaces. d) Temperature variation of the CS and THMC films under one‐sun illumination. e) Frozen droplets spontaneously melt on THMC films under different solar intensities. The red circle indicates melting droplets, while the blue markers represent un‐melted ones. f) Optical images of the cable covered by the THMC film exhibiting reducing and delaying frost formation over time compared with the cable part without the coverage of the film. g) Demonstration of rapid de‐frosting capability of the THMC film adhered to steel sheet. h) Optical images of a droplet continuously impacting the THMC film at −20 °C and bouncing away without the ice formation. i) Infrared optical images of the THMC film adhered to the car model, showing elevated temperature. j) Time‐lapse images of the de‐icing process of the THMC film adhered to the car mode under one‐sun illumination, where formed ice slides off to leave the clean and ice‐free surface.

Conversely, in wet conditions found indoors or outdoors, molecular interfacial forces between PDMS and adhered surfaces are significantly diminished due to the disruption of intermolecular attractions by water molecules. So, the atmospheric pressure exerted by the suction cups serves as the primary source of adhesion in these settings. Besides, the presence of water promotes sealing between the suction cup and surface based on capillary action mechanisms that improve contact integrity. Under the synergistic effect of the above factors, the THMC film shows enhanced adhesion to the surface of a wide array of materials under different humidity conditions compared with that of the pure PDMS substrate (Figure 3i,j). In view of repeated adhesion and detachment, the adhesion cycling test of the THMC film was performed. The THMC film shows good adhesion stability even after 30 cycles, with an obvious reduction in adhesion strength observed (Figure S13, Supporting Information). These results suggest the successful realization of stick‐and‐play functionality, enabling the THMC film to hold great potential for instant adaptability to a wide variety of surfaces in practical scenarios.

### 2.4 Efficient Anti‐Icing/De‐Icing Performance

Benefiting from outstanding hydrophobicity, photothermal performance, and stick‐and‐play functionality, the THMC film is expected to be effective for anti‐/de‐icing applications across a multiplicity of scenarios. It has been reported that the surface microstructure and hydrophobic properties significantly prolong the freezing time of overcooled droplets.^[^ ^9^ ^]^ Thus, the freezing process of the droplets on the THMC film with high hydrophobicity and micro/nanostructures is explored initially. A homemade freezing platform was employed to create a low‐temperature environment (Figure S14, Supporting Information), where the complete freezing process of 20 µL of droplets deposited onto the films was recorded. This simulation condition takes account of the temperature of the ambient air while considering the application scenario where THMC films are adhered, regarding the substrate as an infinite cold source. As shown in Figure 4a and Figure S15 (Supporting Information), the freezing time of droplets on the surfaces of PDMS film, CS film, and THMC film at −20 °C is 106 s, 354 s, and 702 s, respectively (Movie S1, Supporting Information). It is evident that the THMC film exhibits a superior capability of delaying ice formation in comparison with both PDMS and CS film. This could be ascribed to the micro/nanostructured architecture of the THMC film from microscale diatomite and CS nanoparticles. Such a configuration facilitates producing a larger void space during the freezing process, thereby significantly reducing the contact area and increasing thermal resistance. Their synergy diminishes heat transfer efficiency between the droplet and film surface, thereby prolonging the freezing time.^[^ ^17^ ^]^

Considering the contribution of photothermal performance to anti‐/de‐icing ability, the photothermal conversion capability of these films under extremely low temperatures was further evaluated to simulate the real surroundings. As shown in Figure 4d, under one‐sun irradiation at −20 °C, this film rapidly reaches its surface temperature to 7.8 °C, whereas the CS film achieves the maximum temperature to −5 °C under the same conditions. Such a remarkable photothermal effect of THMC films allows them to maintain a surface temperature above the freezing point even under extreme cold conditions, thereby making ice removal through photothermal treatment feasible. As given in Figure 4b and Movie S2 (Supporting Information), under one‐sun irradiation, the melting time of a frozen droplet on the THMC film is about 377 s, whereas that on the CS film is beyond 1800 s, suggesting excellent de‐icing ability of the THMC film. This superior ability might be assigned to the notable thermal management effects observed on the THMC film caused by the cooperation of insulating diatomite and structural design, as discussed above. Assuming the cold substrate and air as unchanging cooling sources, a substantial portion of the energy generated from the light‐to‐heat conversion is exchanged with the surroundings, leading to significant energy loss. Nevertheless, the THMC film possessing low heat transfer efficiency enables a greater proportion of energy to be utilized to interact with supercooled droplets (Figure 4c). Given the falling solar intensity typically below 1 kW m^−2^ of the northern hemisphere in winter, the spontaneous melting processes of frozen droplets on these films were evaluated under different solar intensities toward real scenarios, as shown in Figure 4e. The red circle indicates droplets that are capable of melting, while the blue markers represent those that are unable to be melted within an error range of ±50 Kw m^−^^2^, thereby forming obvious melted and frozen regions. It is observed in Figure 4e that when the temperature and light intensity reach the threshold, the THMC film has the de‐icing ability. So, the remarkable photothermal capabilities make the THMC film function well in thawing frozen droplets even under low solar irradiation. To further validate the adaptability of the THMC film, its anti‐/de‐icing ability was explored when adhered to various surfaces of real objects. As illustrated in Figure 4f, compared to the uncovered one, upon exposure to cold environments for several minutes, the cable surface covered by the THMC film remains partially uncovered by frost. Even after exposure of 2 h, when complete frosting occurs, the covered cable keeps less frost coverage in comparison with the bare one. Besides, such de‐frosting capability of the THMC film was confirmed on various surfaces of engineering materials like glass, stainless steel, and polymer substrates with similar experimental processes. It is noted that the rapid removal of frost from all covered surfaces within minutes was observed after exposure to sunlight (Figure 4g, Figure S17, and Movie S5, Supporting Information). By contrast, the surface without the coverage of THMC films exhibits serious frost coverage.

To demonstrate the instantly sticky anti‐icing performance of the obtained film for real‐world applications, a simulated scenario of icing accretion on the automobile windshield was designed for practical evaluation. The windshield of the car model was divided into two sections, with one half exposed and the other covered by the THMC film, where the anti‐icing experiment was conducted in a cold chamber maintained at −20 °C and approximately 80% relative humidity (Figure S16, Supporting Information). As illustrated in Figure 4h and Movie S3 (Supporting Information), the high hydrophobicity of THMC films enables droplets to consistently rebound from their surface, thereby presenting water spreading and reducing icing risk. In contrast, the exposed section of the car window without the THMC film adheres water droplets and then causes the ice formation. Considering the photothermal icephobic efficacy of the THMC film confirmed above, the corresponding verification experiments were conducted on the car model. Specifically, 100 µL of water droplets were frozen on the car windshield, including THMC film covered part and uncovered one. When they were illuminated by a Xenon lamp for approximately 300 s, the covered part of the windshield could achieve a surface temperature of about 6.1 °C (Figure 4i), which makes the droplet on it melt and slide off. Conversely, the droplet on the uncovered part of the windshield remains frozen (Figure 4j and Movie S4, Supporting Information). These results indicate good de‐icing ability of the THMC film. Additionally, ice adhesion strength has become an indicator for the assessment of anti‐icing properties, which is defined as a bonding strength below 100 kPa for ice detachment from surfaces.^[^ ^40^ ^]^ When under a horizontal shear mode at −15 °C (Figure S18, Supporting Information), ice adhesion strength is measured to be 92.5 kPa, significantly outperforming that observed on glass, stainless steel, and aluminum. The excellent anti‐/de‐icing performance and instantly sticking onto various shape surfaces make the obtained THMC film promising for practical applications in low‐temperature environments.

### 2.5 Durability of the THMC Film

**Figure 5:**
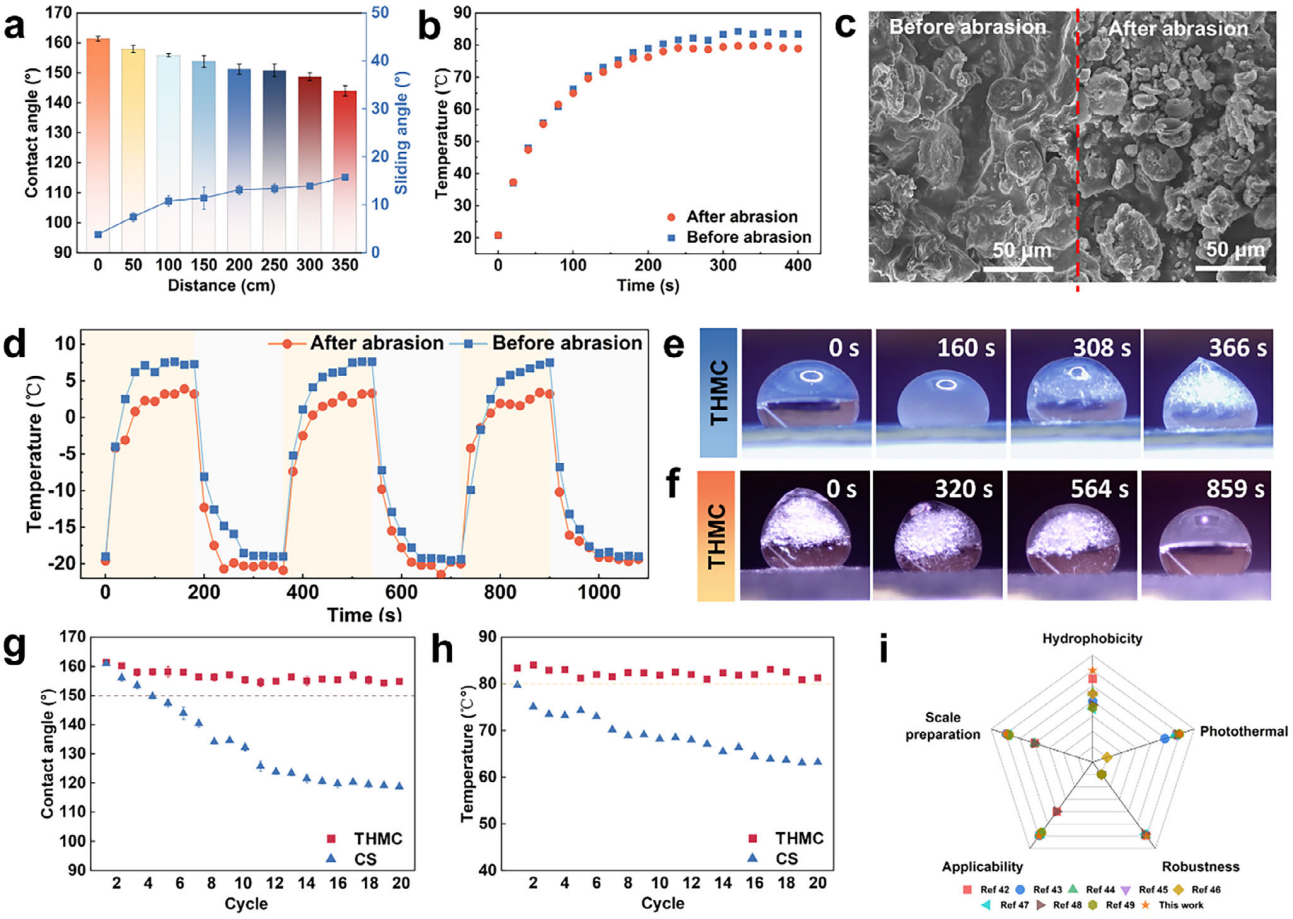
Durability of the THMC film. a) Linear abrasion tests of the THMC film on 1000 grit sandpaper with a load of 5.6 kPa. b) Temperature‐time curve at room temperature under a solar irradiation intensity of 1 kW/m^2^ after linear abrasion. c) SEM image of the THMC film before and after experiencing 300 cm of linear abrasion. d) Temperature‐time curve at −20 °C under a solar irradiation intensity of 1 kW/m^2^ after linear abrasion. e) Icing delay process of droplets on the surfaces of the THMC film after linear abrasion. f) Melting process of a frozen droplet on the surfaces of the THMC film after linear abrasion. Wetting behavior g) and surface temperature h) of THMC and CS films within 20 icing/de‐icing cycles. i) Comprehensive comparison of photothermal icephobic films with previous reports.

In real world, ice coverage and accumulation usually occur under harsh environmental conditions, which poses a high requirement for the stability of the THMC film. As designed, micro/nanostructures are constructed for the superhydrophobicity of the THMC film, which is adverse for its long‐term service, as demonstrated by previous reports.^[^ ^41^ ^]^ To overcome this issue, THMCs with armor architecture and hierarchical self‐similarity structure are designed and balanced, whose high hydrophobicity and photothermal effect have been substantiated above. However, the durability of these performances induced by structural damage is unclear, which is critical for practical applications. Thus, it is necessary to evaluate the durability of the THMC film. After experiencing common usage conditions involving random repeated friction, scraping, and rubbing, the THMC film retains its high hydrophobicity (Figure S19 and Movie S6, Supporting Information). Additionally, the mechanical robustness of the THMC film was evaluated using the sand‐fall method (Figure S20, Supporting Information). After exposure to 160 g of sand abrasion, the superhydrophobicity of the film almost maintains, indicating substantial mechanical durability. When suffering from mechanical linear abrasion tests using 1000‐grit sandpaper at a load of 5.6 kPa across 300 cm, the abraded THMC film still exhibits high hydrophobicity with the CA of 148.7° (**Figure** **5**a). Except for changeless surface wetting behavior, the photothermal effect of the THMC film seems to be unchanged when exposed to sunlight at ambient room temperature, as supported by Figure 5b, where the surface temperature of the abraded film exceeds 80 °C under one‐sun irradiation at room temperature. This should be ascribed to the fact that the surface morphology of the THMC film exhibits similarities to its original state (Figure 5c). After wearing test, a considerable portion of CS and diatomite were lost from the surface, yet THMC remained residual (Figure S21b,c, Supporting Information), enabling the surface to retain sufficient microstructures and photothermal conversion materials to enable its photothermal effect and hydrophobic property. Compared to CS films, THMC films exhibit less mass loss during abrasion (Figure S21d, Supporting Information), indicating a superior advantage in resistance to wear. The enhanced abrasion resistance, when compared to pure PDMS, originates from the protective effect afforded by the diatomite framework. This characteristic makes the THMC film suffering from 300 cm linear abrasion still maintain a surface temperature above 0 °C in a −20 °C environment (Figure 5d). When the surface temperature is less than 0 °C, ice formation would be delayed. As demonstrated in Figure 5e,f, upon exposure to sunlight, the THMC film could still delay ice formation above 6 minutes and completely thaw frozen droplets within 15 min (Movie S7, Supporting Information).

Owing to exceptionally low contact angle hysteresis, superhydrophobic surfaces could rebound droplets effectively from their surfaces, thereby dynamically inhibiting ice formation by minimizing contact time.^[^ ^5^ ^]^ Consequently, the study on the liquid drop rebounding on the THMC film after mechanical damage is able to further assess the anti‐icing ability. When droplets impact the abraded film, it could rapidly rebound from this film despite a slight increase in contact time (Figure S22, Supporting Information). This indicates the maintenance of both strong hydrophobicity and excellent anti‐icing ability after mechanical abrasion. Additionally, ice adhesion strength on the THMC film was measured after multiple de‐icing cycles (Figure S23, Supporting Information). It is observed that the ice adhesion strength after 20 cycles is about 147.3 kPa, indicating sustainability after repeated use. Moreover, compared with the CS film after multiple de‐icing cycles, the THMC film displays significant disparities in hydrophobicity and photothermal conversion efficiency (Figure 5g,h). With de‐icing cycles increasing, the THMC film maintains a CA of water droplet exceeding 150° on its surface while achieving surface temperatures above 80 °C under sunlight exposure even after 20 cycles. To demonstrate such capabilities of the THMC film, a comprehensive comparison of this work with the ice‐repellent films reported recently was conducted, as given in Figure 5i.^[^ ^42^, ^43^, ^44^, ^45^, ^46^, ^47^, ^48^, ^49^ ^]^ The THMC film exhibits significant advantages in terms of photothermal performance, hydrophobic properties, and functionality (for more details, see Table S1 in the Supporting Information). These results confirm that the THMC film displays exceptional durability, showing great promise for practical anti‐/de‐icing applications.

## 3 Conclusion

In summary, inspired by the hollow framework structure of lotus seedpods, we have proposed a durable THMC with armor architecture and hierarchical self‐similar structure by incorporating nanoscale CS into porous microscale diatomite. The resultant PDMS films embedding THMC are endowed with stick‐and‐play functionality by constructing a sucker array on its reverse surface via a template method. The obtained film demonstrates effective liquider repellency across a range of substances, showing a CA above 150° and SA below 10°. Owing to the surface thermal accumulation effect induced by bioinspired THMC based on the synergistic thermal management of the insulating diatomite and photothermal CS within the hierarchical structure, the surface temperature of the THMC film can attain 84.2 °C at room temperature under one‐sun irradiation. Moreover, the stick‐and‐play functionality enables the film to adhere to a wide range of surfaces. The film can also be adjusted through lower strength for easy peeling and transfer. When adhered to various surfaces of engineering materials, the THMC film demonstrates exceptional anti‐/de‐icing capabilities, effectively delaying ice formation by 702 s while facilitating rapid ice and frost removal under one sun illumination condition. Even after undergoing 300 cm of linear abrasion, the THMC maintains effective photothermal anti‐/de‐icing performance, indicating its remarkable robustness. The design of micro‐units combining this photothermal material with thermal insulation frameworks can effectively enhance the performance of the device while exhibiting high scalability. Meanwhile, the successful preparation of stick‐and‐play functionality films also holds significant potential for large‐scale fabrication and large‐area applications. We believe that this constructing strategy built on the thermal management and structure design positively innovates anti‐icing technology under low‐light conditions and addresses critical issues related to the durability and scalable production of relevant devices.

## 4 Experimental Section

### Preparation of Photothermal Hydrophobic Micro‐Cells (THMC)

Initially, 1 g of the collected candle soot was weighed and dispersed in 50 g of ethanol using a sonic disperser for a duration of 60 min. Subsequently, 3 g of diatomite was added into the dispersed solution and thoroughly mixed. The resulting mixture was vacuum‐dried at 70 °C to yield a homogeneous product. Finally, the obtained black particles were filtered through a 300 mesh sieve for further use.

### Preparation of THMC Film

A uniform layer of PDMS prepolymer was applied to a square mold (with groove dimensions of 35 mm × 35 mm × 1 mm, fabricated via 3D printing using ABS resin). After the curing of the prepolymer at 60 °C for 9 to 10 min, a semi‐polymerized PDMS film was formed. Then, 0.1 g of THMCs particles were evenly distributed across the surface of the PDMS film, which was further cured for an additional 10 min before removal. Subsequently, 0.2 g of PDMS was dispersed in a solution containing 100 mL of n‐heptane and sprayed onto the surface of the PDMS film. After being cured for 2 h, the resultant film was carefully peeled off from the mold to yield the THMC film.

### Sample Characterization

The surface morphology was analyzed using a field emission scanning electron microscope (SEM, SU8020, Hitachi, Japan). Contact angles and sliding angles were captured with a video‐based optical contact angle measurement system (DSA100E, KYUSS, Germany), taking measurements from various random positions on the surface more than five times. The UV‐vis spectrum was obtained using a UV‐3600 visible spectrophotometer (Shimazu, Japan) over the wavelength range of 200–2500 nm. Surface temperature on the THMC film was measured with an infrared thermal imager (FLIR E6, USA). Simulated sunlight for photo‐thermal conversion and ice‐removal experiments was generated by a Xenon light source (MC‐PF300, Merry Change, China). Thermal conductivity was measured using the Hot Disk method (TPS2500S, Sweden). A Fourier infrared spectrometer (FTIR) was used to characterize the infrared emission spectra of the sample at 2.5–20 µm band. Testing was performed no less than three times, depending on the variability of the results and the desired level of precision.

## Conflict of Interest

Y.Z., X.X., and H.C. are inventors on a nonprovisional granted patent related to this work filed by China National Intellectual Property Administration (No. ZL202411119947.4). The authors declare no other competing interests.

## Supporting information



Supporting Information

Supplemental Movie 1

Supplemental Movie 2

Supplemental Movie 3

Supplemental Movie 4

Supplemental Movie 5

Supplemental Movie 6

Supplemental Movie 7

## Data Availability

The data that support the findings of this study are available from the corresponding author upon reasonable request.
