# Supplementary material for: Robust Stick‐and‐Play Photothermal Icephobic Film with Bioinspired Insulation Cells
Source: Adv Sci (Weinh). 2025 Mar 16;12(18):2502687. doi: 10.1002/advs.202502687 (PMC12079502; doi:10.1002/advs.202502687)
Supplement: Supplementary file 1 — Supporting Information [file ADVS-12-2502687-s006.pdf]

ADVANCED SCIENCE
Open Access

Supporting Information

for *Adv. Sci.*, DOI 10.1002/advs.202502687

Robust Stick-and-Play Photothermal Icephobic Film with Bioinspired Insulation Cells

*Xiaohu Xia, Haotian Chen, Yiming Wang, Haidong Yu, Bingsuo Zou and Yabin Zhang**

Supporting Information

Robust stick-and-play photothermal icephobic film with bioinspired insulation cells

Xiaohu Xia, Haotian Chen, Haidong Yu, Yiming Wang, Bingsuo Zou, Yabin Zhang*
State Key Laboratory of Featured Metal Materials and Life-cycle Safety for Composite Structures, Guangxi Key Laboratory of Processing for Non-ferrous Metals and Featured Materials, MOE Key Laboratory of New Processing Technology for Nonferrous Metals and Materials, Guangxi University, Nanning 530004, P. R. China

Contents:

1. Supplementary Methods

1.1 Materials

1.2Thermal response test

1.3 Self-cleaning experiment

1.4 Calculation of photothermal conversion efficiency

1.5 COMSOL simulation of thermal filed in THMC film

1.6 Adhesive performance evaluation

1.7 Anti-icing and de-icing tests

1.8 Simulated de-icing and de-frosting experiments

1.9 Mechanical durability experiments

2. Supplementary Figure Legends: Figure S1-S22

3. Supplementary Table: Table S1

4. Supplementary Movies: Movie S1-S7

1. Supplementary Methods

1.1 Materials

Candle soot (CS) was collected by combusting standard white candles (TaTannice Company, China). The diatomite (Shanghai Aladdin Bio-Chem Technology Co., LTD.) with a median particle size of 20 μm was pre-filtered through a 300-mesh sieve to remove large particle impurities before use. Polydimethylsiloxane (PDMS) film was synthesized using Sylgard 184 (Dow Corning Co., LTD.) at a weight ratio of 10:1 between the base and curing agent. Sodium hydroxide (NaOH) and n-heptane (98%) were procured from Aladdin Company, China. Hydrochloric acid (HCl), acetone, and ethanol were purchased from Blue Sky Experimental Equipment Co., Ltd., Nanning, China.

1.2 Thermal response test

The thermal response characterization was conducted under Xenon lamp irradiation, with precise control of light intensity through power regulation of the Xenon source. Temperature evolution was simultaneously monitored using both contact thermal sensors and non-contact infrared thermal imaging, ensuring comprehensive thermal profiling.

1.3 Self-cleaning experiment

For the self-cleaning evaluation, samples were mounted on a platform with a 30-degree inclination angle. Uniformly distributed quartz sand was applied to the sample surface, followed by controlled water droplet deposition. The entire self-cleaning process was quantitatively analyzed through high-speed camera monitoring, allowing for detailed observation of the dynamic behavior.

1.4 Calculation of photothermal conversion efficiency

The photo-to-thermal conversion efficiency of the material, η, which was defined as the ratio of the heat generated by the photothermal conversion of the material to the input of solar power, and the calculation formula is as follows:

$$\eta = \frac{Q}{q \times A}$$

where Q is the heat generated by the light-to-heat conversion of the material, q is the input of solar power density (q = 100 mW/cm^2), and A is the surface area (A = 9 cm^2) of the photothermal anti-icing material.

When the surface temperature tends to be stable, the heat generated by the light-to-heat reaches a balance with the heat dissipation to the surrounding environment, which can be described as:

$$Q = Q_S = h \times A \times (T_{sub} - T_{surr})$$

where Q_s is the heat dissipation to the surrounding environment, h is the average convective heat transfer coefficient of the materials, T_{sub} is the substrate temperature tends to be stable, and T_{surr} is the environment temperature. The convective heat transfer coefficient h can be calculated by the following formula:

$$N_u = \frac{h \times L}{\lambda}$$

where N_u is the Nusselt number, L is the characteristic length of the heat transfer region (L = 3 cm), λ is the thermal conductivity coefficient of the fluid (here is air). The calculation formula of Nusselt number in this experiment is as follows:

$$N_u = 0.54 \times (G_r \times P_r)_m^{\frac{1}{4}}$$

where G_r is The Grashof number, P_r is the Prandtl number, the subscript m represents the qualitative temperature using the arithmetic mean temperature of the boundary layer (T_m = (T_{sub} + T_{surr})/2), and the calculation formula of G_r, P_r in this experiment is as follows:

$$G_r = \frac{g\alpha_v \Delta T L^3}{v^2}$$

$$P_r = \frac{v}{a}$$

where g is the acceleration of gravity (g = 9.8 m/s^2), α_v is the volume expansion coefficient (for an ideal gas, α_v = 1/T), ΔT is the average temperature difference between the surface and air, v is the viscosity coefficient, a is the thermal diffusivity.

	CS	THMC		CS	THMC
**Ts (°C)**	79.7	84.2	**λ×10^{-2} W/(m·K)**	2.9	2.9
**ΔT (°C)**	58	63.5	**ν×10^6 (m^2/s)**	18.2	18.3
**Tm (°C)**	50.7	52.45	**a×10^6 (m^2/s)**	26.1	26.4
**T (K)**	323.85	325.6	**αv×10^3 (K^{-1})**	3.09	3.07
**Pr**	0.697	0.693	**Gr×10^{-4}**	14.3	15.4
**Nu**	9.59	9.76	**h (W/m^2·K)**	9.27	9.43
**Q (mW)**	483.9	538.9	**η (%)**	53.8	59.9

1.5 COMSOL simulation of temperature distribution in THMC film

A finite element model (FEM) was employed to compare the thermodynamic differences between two films under irradiation. The model includes two systems: one is a PDMS patch with a top layer mixed with 50 nm carbon particles, and the other is a similarly sized PDMS patch with a top layer mixed with SiO_2 particles, whose surfaces are coated with carbon particles, as illustrated. The primary heat source in this model is solar irradiation, simulated using the External Radiation Source feature. The incident solar radiation is directed perpendicularly to the surface, with a solar flux of 1000 W/m^2. All ambient surfaces in the model are considered in the solar loading calculation, and shadowing effects are included. The sun, with a temperature of approximately 5800 K, primarily emits short-wavelength infrared and visible light at wavelengths shorter than 2.5 μm. The fraction of this short-wavelength solar radiation absorbed by the materials is quantified by their solar absorptivity. The model accounts for the wavelength dependence of emissivity to reflect differing emissivity in various wavelength bands.

1.6 Adhesive performance evaluation

Adhesion tests on solid materials were conducted using a custom-built device that incorporated a load cell for force detection and a universal connector for securing the tested substrate. The load cell, which was employed to apply preload and measure adhesion force, was mounted on a movable platform. Controlled downward displacement enabled contact between the solid substrate and the sample, thereby increasing the loading force, while upward movement facilitated the

detachment of the substrate from the sample.

1.7 Anti-icing and de-icing tests

An experiment to assess anti-icing performance was conducted in a custom-designed cold chamber. The temperature of the cooling platform within the chamber was maintained at -20°C by circulating a mixture of glycol and water, which was cooled using a refrigeration system. Samples were positioned on the cooling platform with 20 μL droplets of water applied to their surfaces. The freezing process of these droplets on the samples was simultaneously recorded using an industrial electron microscope (WO-HD227, AOSVI, China), a T-type thermocouple, and an infrared thermal imager (FLIR E6, USA). The anti-icing efficacy of the samples was evaluated by measuring the delay time before water droplets completely freezing.

In the ice adhesion strength experiment, a force sensor (Imada ZP-100N) connected to a computer was employed to measure the ice adhesion strength at -20°C. Initially, the specimen was carefully positioned on a petri dish, followed by the addition of 2 mL of water. Subsequently, the water-filled petri dish was frozen alongside the specimen for 2 hours to ensure complete solidification. Finally, the probe of the force sensor was activated to apply pressure on the ice column at a rate of 0.5 mm/s during testing. The software recorded both force and displacement curves necessary for separating the ice column. The ratio of peak force to cross-sectional area provided an accurate measurement of ice adhesion strength obtained in this study. The precision of force measurements reached up to 0.01 N, with an ice column cross-section measuring 10×10 mm. Furthermore, each sample's ice adhesion strength was measured at least three times.

The photothermal de-icing experiment was conducted on a cooling platform under the condition of xenon lamp simulating sunlight. The droplets on the sample were placed in the freezer for at least 2 hours to ensure complete freezing. Then, the xenon lamp was turned on, and the defrosting process was recorded using an industrial electron microscope. The effect of linear wear on the surface's photothermal defrosting ability was evaluated by studying the melting time of the ice on the surface before and after linear wear.

1.8 Simulated de-icing and de-frosting experiments

The experiment was carried out in a custom-designed cooling chamber, with the ambient temperature maintained at -20°C. Humidity levels were regulated at 80%±5% using a humidifier and humidity sensor. In the de-icing experiment, a volume of 1 mL of water was completely frozen on both the THMC film and the bare model surface, after which the samples were irradiated with an Xe lamp (irradiation intensity equivalent to 1 sun) serving as the light source. The melting process of the surface droplets was documented using a digital camera (R50, Canon, Japan), and the duration from the initiation of irradiation to when the frozen droplets detached from the sample model surface was utilized as a criterion for assessing de-icing performance. In the de-frosting experiments, samples were subjected to freezing for 3 hours in a cooling chamber to ensure a uniform layer of frost formation. Subsequently, the samples were irradiated with xenon lamps (irradiation intensity equivalent to 1 sun), and the defrosting process was documented using a digital camera. The criterion for evaluating defrosting performance was defined as the duration from the initiation of irradiation until the complete removal of frost from the sample surface.

1.9 Mechanical durability experiments

In the linear wear test, the measured surface was subjected to abrasion under a pressure of 5.6 kPa using 1000-grit sandpaper. A wear cycle was defined as a linear distance of 50 cm. In the sand abrasion experiment, the sample underwent grinding with quartz sand, maintaining a distance of 40 cm between the sample surface and the sand container, while positioned at an angle of 45°. During each grinding cycle, 20 g of quartz sand was uniformly applied onto the sample surface.

2. Supplementary Figure Legends

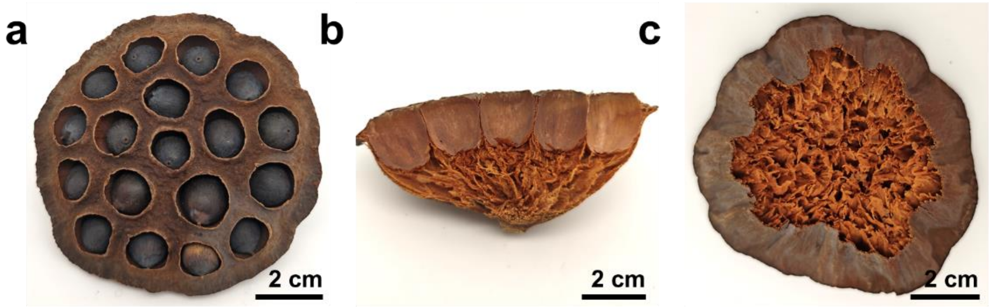

Figure S1. a) The optical photograph of the exterior of a dried lotus pod depicts its composition of lotus seeds and seed pods. b) The lateral cross-section and c) transverse cross-section of the lotus pod exhibits numerous hollow structures pervading throughout the structure.

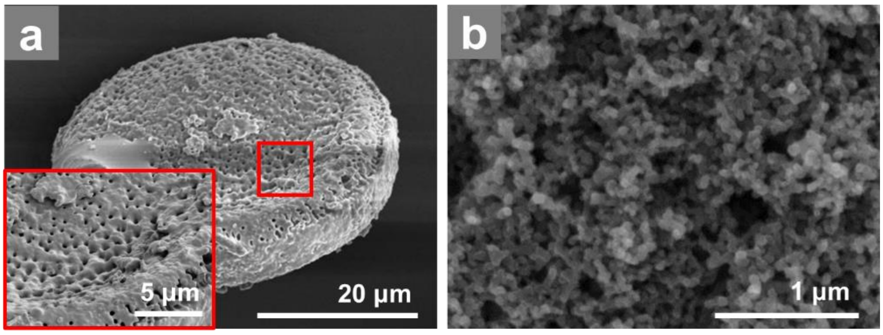

Figure S2. SEM images of a) diatomite and b) candle soot.

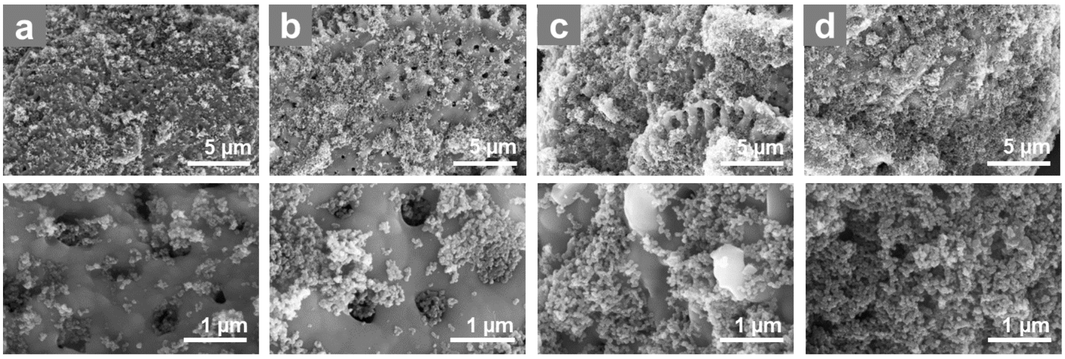

Figure S3. High- and low-magnified SEM images illustrating the morphology of the THMCs with different mass ratios of candle soot to diatomite in a) 1:1, b) 1:2, c) 1:4, and d) 1:5, where micro-cells are distributed onto the diatom.

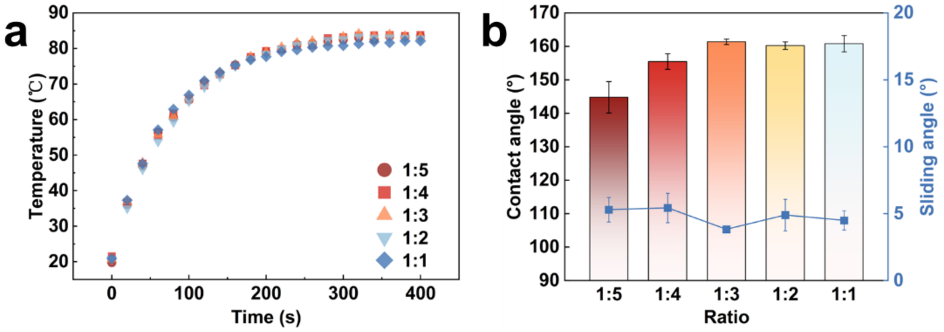

Figure S4. a) Contact angles and sliding angles of the THMC films with different amounts of microcells. b) Temperature-time curve of the THMC films with different amounts of microcells under one sun irradiation.

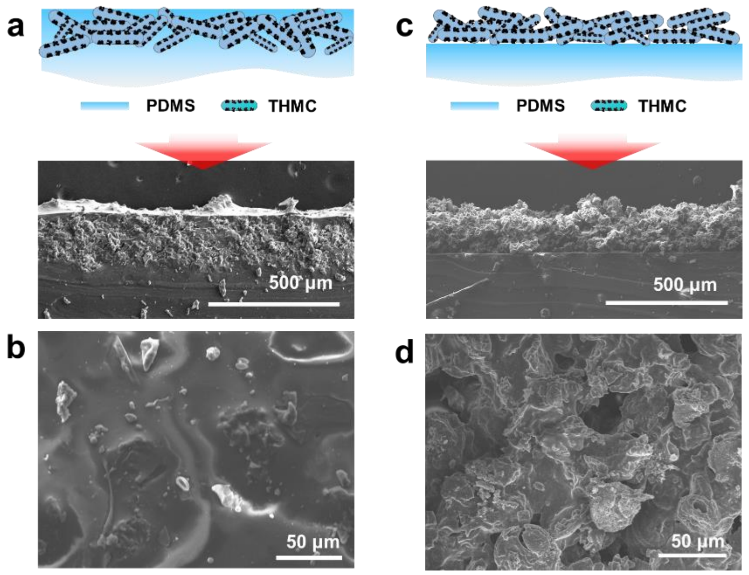

Figure S5. The polymerization state of PDMS significantly influences the performance characteristics of the films. Schematic diagram and SEM images of the THMC film with THMCs submersed into the PDMS substrate (a) and exposed on the surface of the PDMS substrate (c). SEM images of the THMC film surface with THMCs exposed on the surface of the PDMS substrate (b) and exposed on the surface of the PDMS substrate (d).

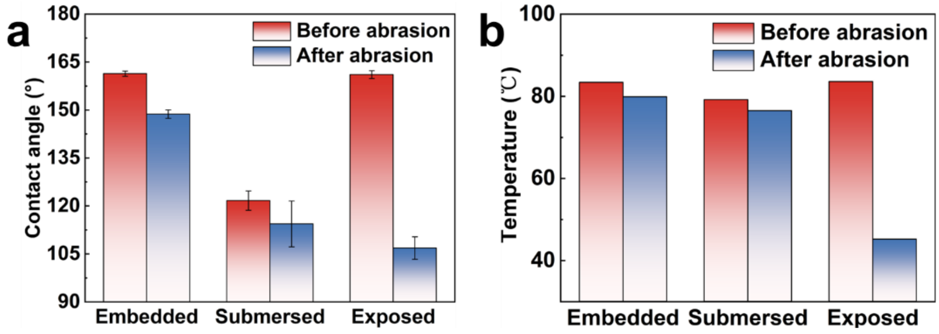

Figure S6. Performance comparisons of the films with different states. a) Contact angles of the films before and after abrasion. b) Maximum temperatures reached by the films before and after abrasion.

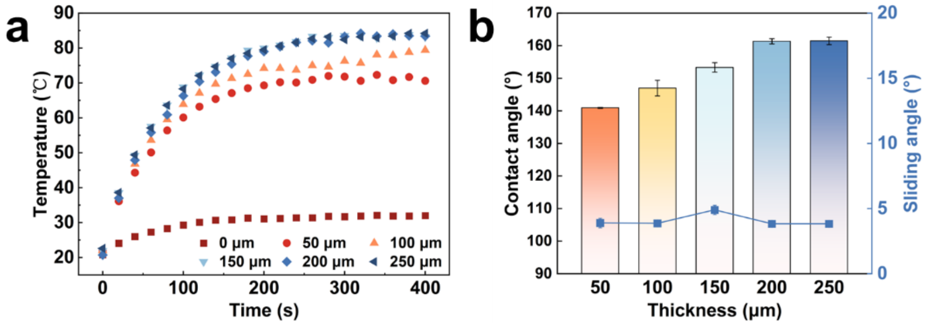

Figure S7. a) Temperature-time curves of THMC films with different spraying thicknesses under one sun irradiation. b) Contact angle and sliding angle for THMC films with different spraying thicknesses.

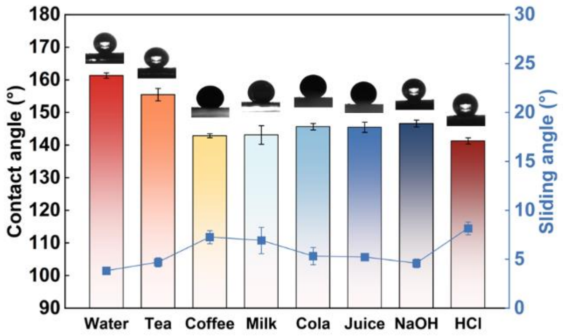

Figure S8. Contact and sliding angles for various liquids on the THMC film, highlighting its resistance to multiple potential contaminants.

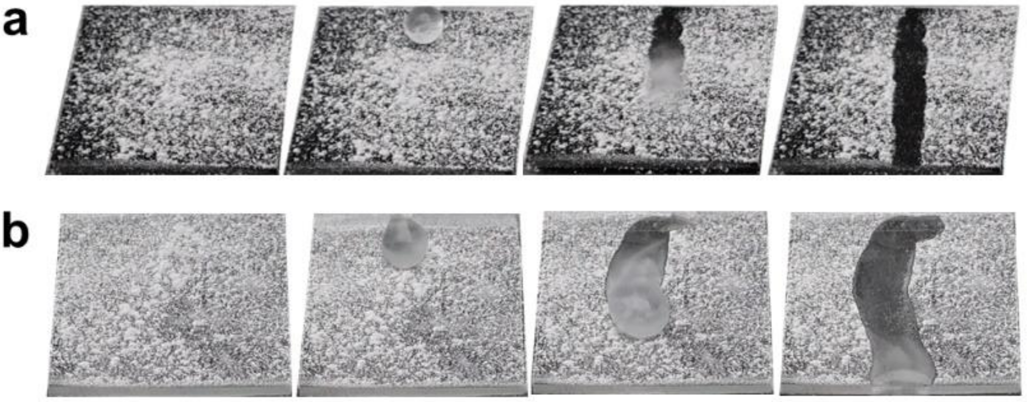

Figure S9. a) Self-cleaning ability of dust by water droplets on the THMC film. b). Water droplets that slide off the PDMS film without self-cleaning effect.

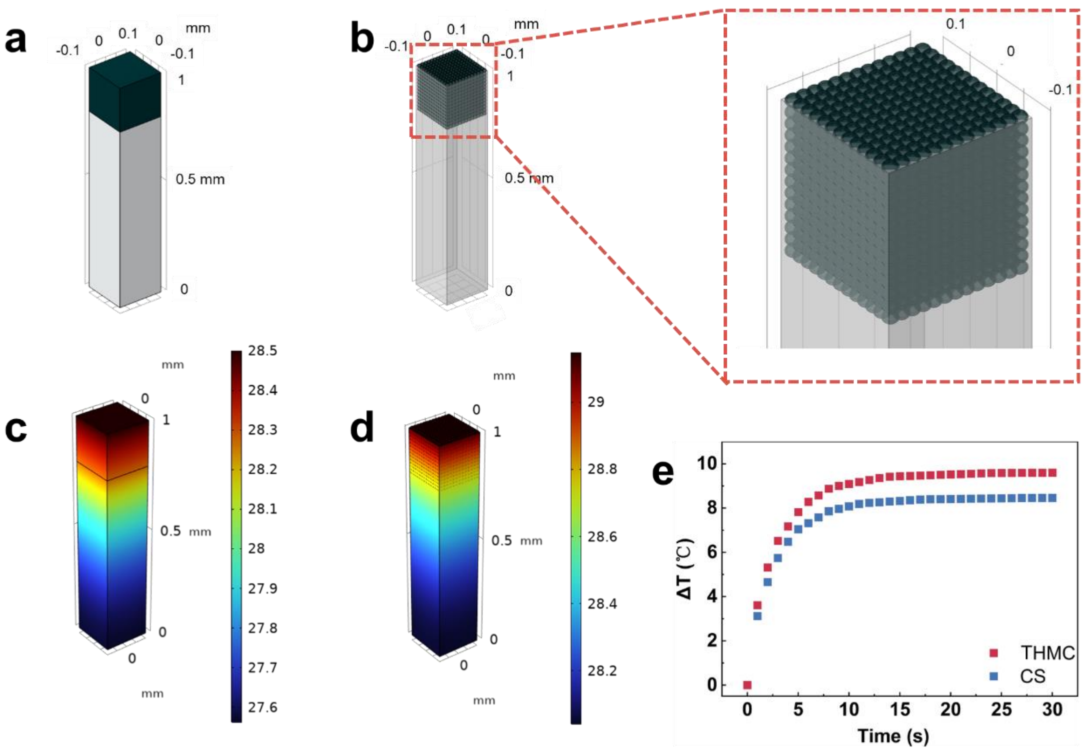

Figure S10. COMSOL model of a tiny unit taken from the film with micro-cell structure. a) CS film and (b) THMC film. Simulated temperature ramping process of (c) CS film and (d) THMC film, where the thermal accumulation effect on the surface of a tiny unit from the THMC film is evident, suggesting that many units would result in high surface temperature of the THMC film. e) Surface temperature variation curves of the CS and THMC film over time in the simulation.

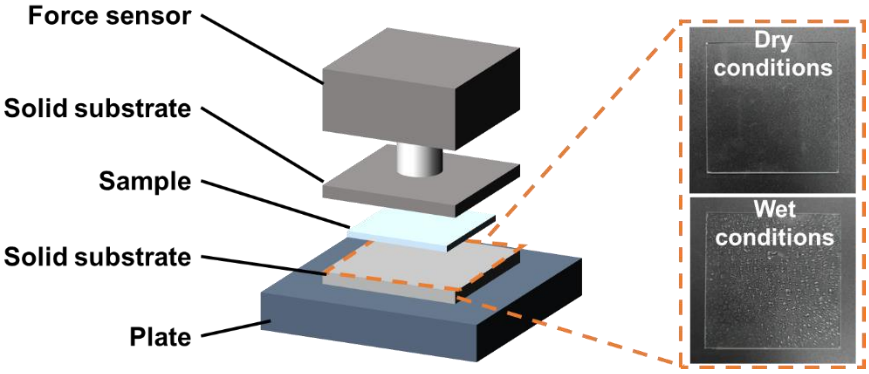

Figure S11. A customized device for measuring the adhesion parameters of suction cup arrays on the THMC film.

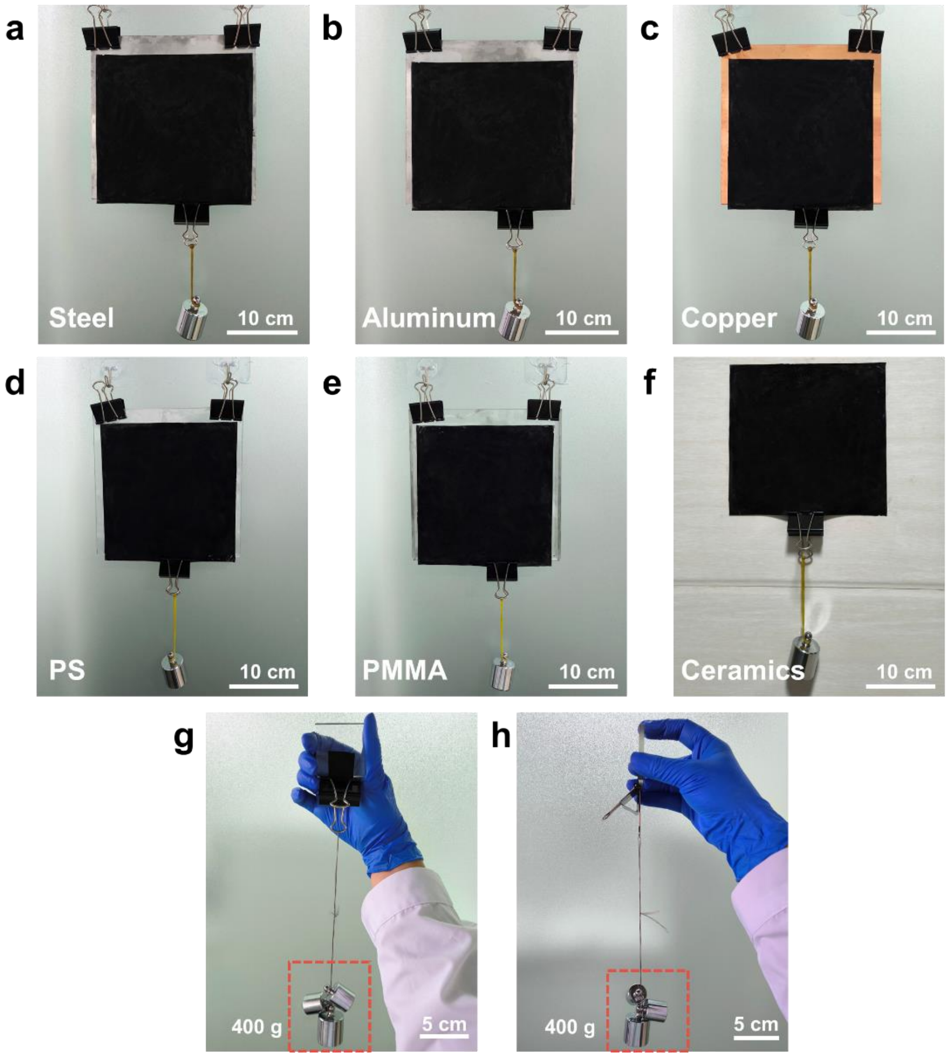

Figure S12. Optical photos depicting the adhesion of the THMC film onto various material surfaces: a) steel, b) aluminum, c) copper, d) Polystyrene (PS), e) Polymethyl Methacrylate (PMMA), and f) ceramics, evaluated by the constant weight of 200 g. g-h) Optical images of

THMC film showing remarkable tangential adhesion to glass, maintaining adherence even under a load of 400 g, with an effective adhesive area of approximately 20 mm × 20 mm.

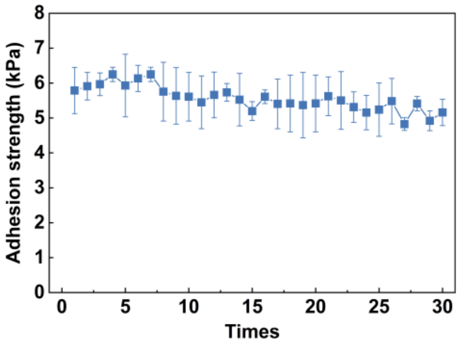

Figure S13. Cyclic adhesion strength of the THMC film, suggesting that there is no significant decrease in adhesive strength after 30 cycles.

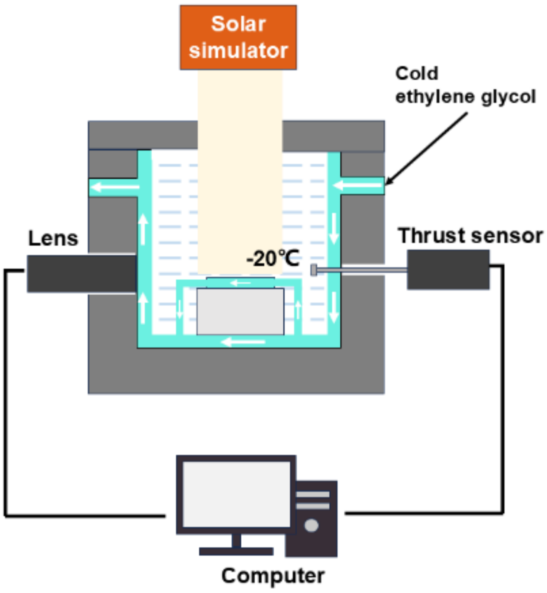

Figure S14. Schematic diagram of a customized equipment used to establish low-temperature environments for droplet freezing experiment.

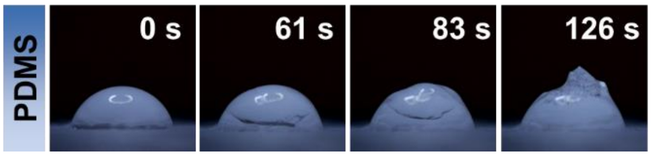

Figure S15. Icing delay process of droplets on the surfaces of the PDMS film.

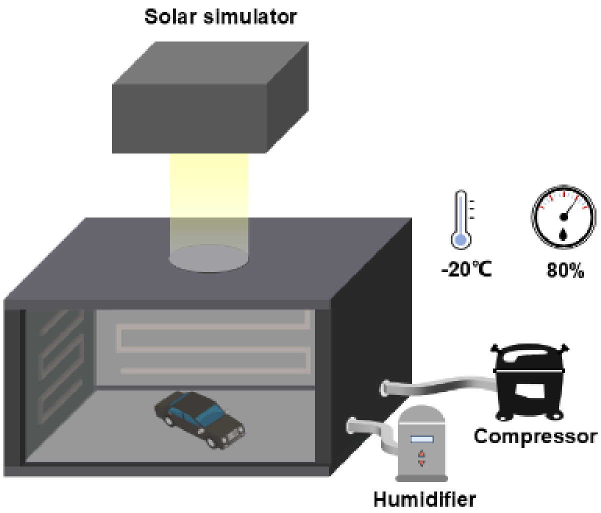

Figure S16. Schematic diagram of customized equipment used to simulate low-temperature environments for simulated the practical application of THMC films.

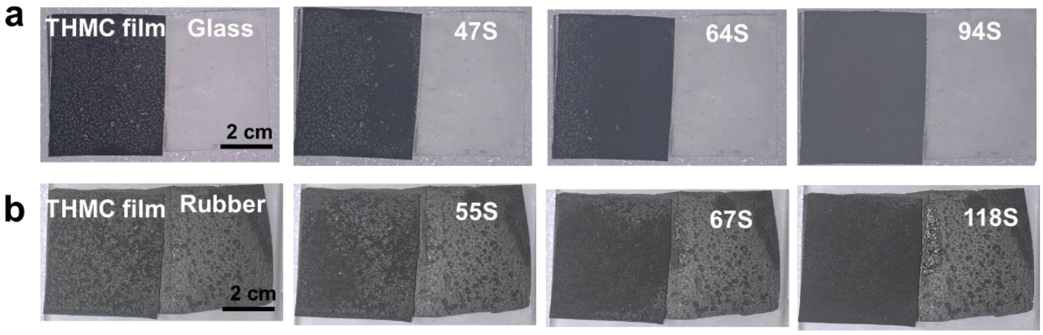

Figure S17. Time-lapse snapshots taken during the de-frosting processes on RHMC film adhering to glass (a) and rubber (b) substrates.

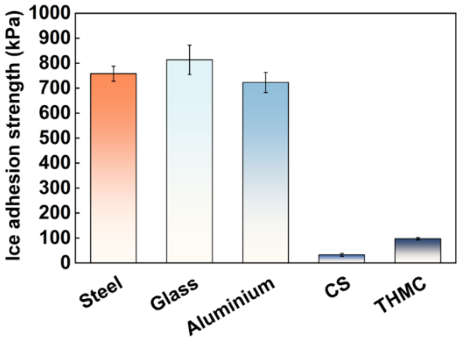

Figure S18. Ice adhesion strength of THMC film and other materials, suggesting good icephobic ability of the THMC films.

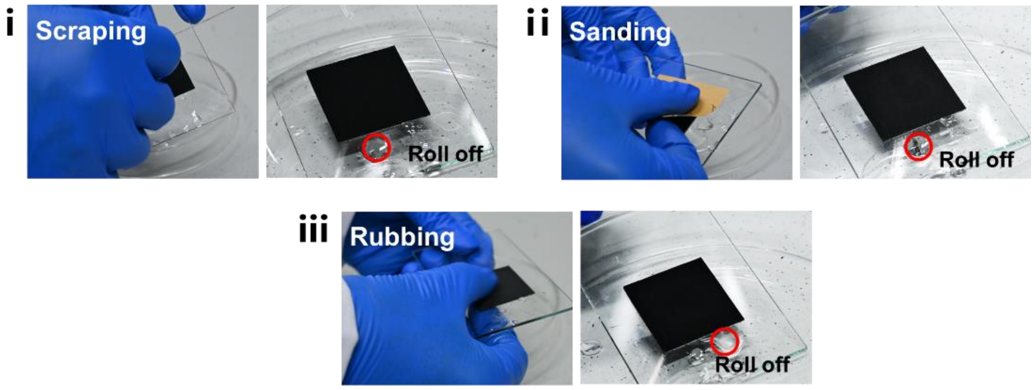

Figure S19. Photographs of the superhydrophobic behaviors of the THMC film after various mechanical damages.

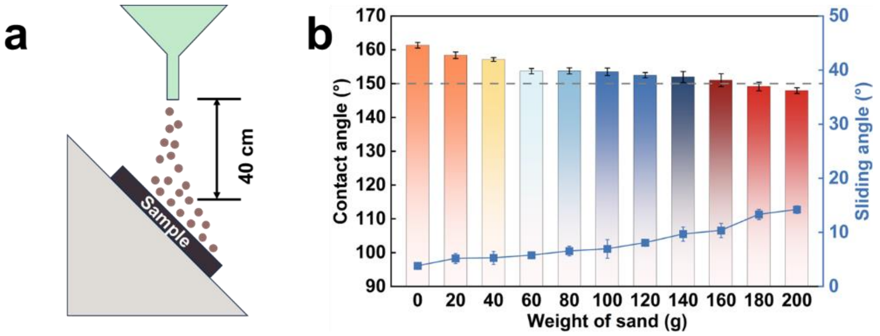

Figure S20. a) Schematic illustration of the sand impact onto the THMC film. b) Surface wettability changing curves with the sand falling weight. The contact angles of THMC film decrease and the sliding angles increases with falling weight, indicating that the superhydrophobicity of the THMC film changes slightly after 180 g sand abrasion.

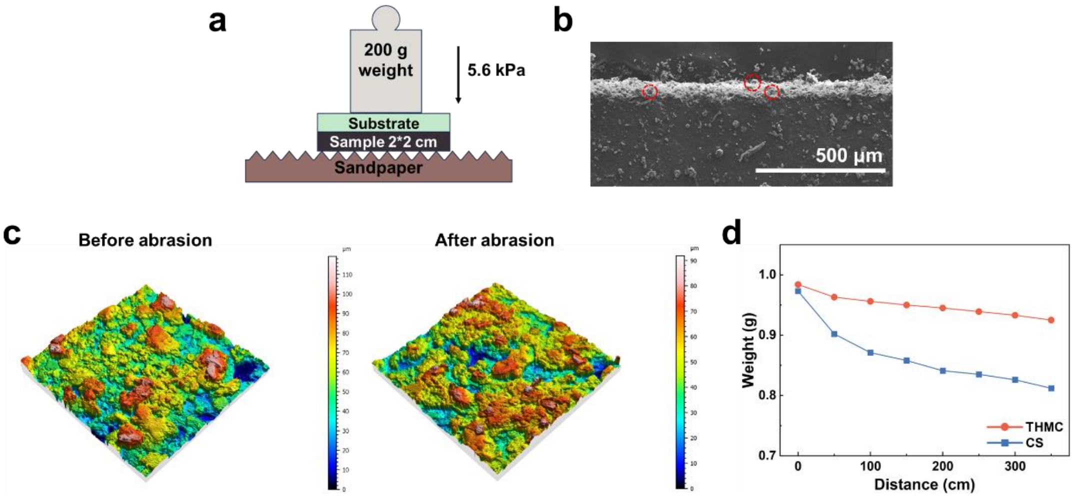

Figure S21. a) Schematic of the THMC film suffering from sandpaper abrasion. b) SEM image of the THMC film after experiencing 300 cm of linear abrasion. c) 3D profile scanning images of the THMC film before and after wear testing. d) Changes in the weight of the film during the wear experiment.

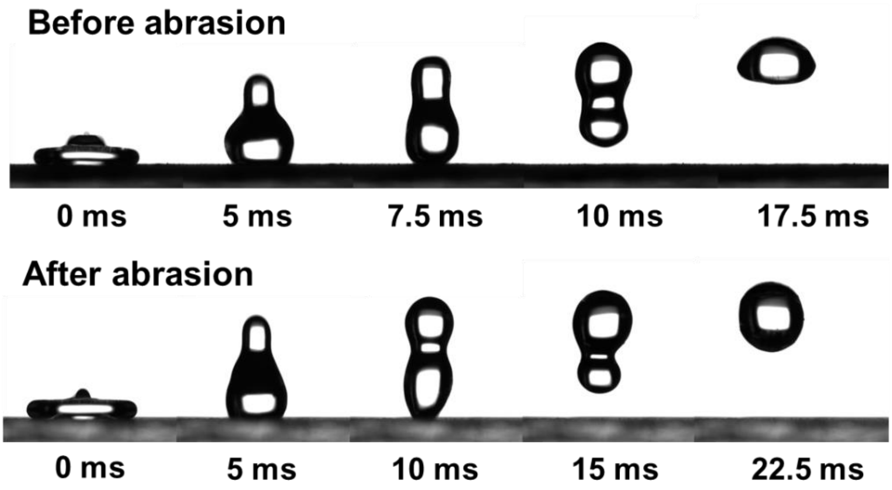

Figure S22. Bouncing behavior of droplets on the surface of the THMC film before and after abrasion.

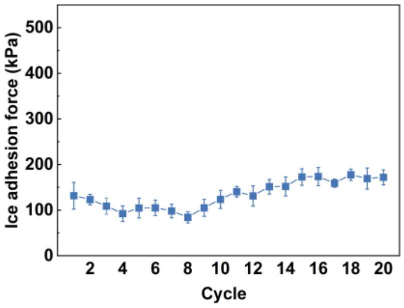

Figure S23. Ice adhesion strengths on THMC film surfaces within 20 icing/de-icing cycles.

3. Supplementary Tables

Table S1. Comparison of photothermal icephobic films with previous reports.

Material	Structures	Contact angle	Photothermal temperature (Environment temperature)	Photothermal temperature during freezing (Cold table temperature/air temperature)	Ref.
**Candle soot, diatomite, and PDMS**	**Porous hierarchical structure**	**161°**	**84.2 ℃ (20 ℃)**	**7.8 ℃ (-20 ℃/-20±5 ℃)**	**This work**
PDMS/Ni@Ti3C2Tx	Eyelash-like Microstructure array	154.7	ΔT=56.7 ℃	10 ℃ (-20 ℃/room temperature)	[42]
CNT, Ti3C2Tx, and PDMS	Particle protrusion	128°	61.3 ℃ (24 ℃)	2.1 ℃ (-10 ℃/room temperature)	[43]
Iron powder, candle soot, and PDMS	Double layered porous structure	>150°	75 ℃ (30 ℃)	30 ℃ (-5 ℃)	[44]
PMMA@MWCNTs membrane	Porous structure	155°	80.9 ℃ (37 ℃)	9.4 °C(-30 ℃/room temperature)	[45]
thermoplastic polyurethane@carbon nanotube/graphene nanoplatelet	Fiber structure	/	74.2 °C (30 ℃)	20 °C(-10 ℃/room temperature)	[46]
MWNTs, shape memory epoxy resin, PDMS	Irregular micro protrusions	153.9°	/	2.1 ℃ (-10 ℃/room temperature)	[47]
Titanium nitride and polytetrafluoroethylene	Separated nanorod shapes	156°	60 °C (25℃)	/	[48]
Nano-scale carbon black co-electrospinning-electrospray	Fiber and protrusion structure	160°	59.8 °C (28 ℃)	/	[49]

4. Supplementary Movies: Movie S1-S7

Movie S1: Icing delay process of droplets on the surface of different films.

Movie S2: Melting process of frozen droplets on the surface of different films.

Movie S3: Droplets bouncing off the THMC film surface at low temperatures.

Movie S4: Freezing droplets sliding off the surface of the THMC film under light illumination.

Movie S5: De-frosting of the THMC film surface under light illumination.

Movie S6: Durability test involving random repeated abrasion, scraping, and rubbing.

Movie S7: Icing delay and melting process of droplets on the surfaces of the THMC film following linear abrasion.
